# Supplementary material for: Efficient and precise single-cell reference atlas mapping with Symphony
Source: Nat Commun. 2021 Oct 7;12:5890. doi: 10.1038/s41467-021-25957-x (PMC8497570; doi:10.1038/s41467-021-25957-x)
Supplement: Supplementary file 1 — Supplementary Information [file 41467_2021_25957_MOESM1_ESM.pdf]

Supplementary Information

for

Efficient and precise single-cell reference atlas mapping with Symphony

Joyce B. Kang[1-5], Aparna Nathan[1-5], Kathryn Weinand[1-5], Fan Zhang[1-5], Nghia Millard[1-5], Laurie Rumker[1-5], D. Branch Moody[3], Ilya Korsunsky[1-5]*, Soumya Raychaudhuri[1-6]*

* Correspondence to: I.K. (ikorsunskiy@bwh.harvard.edu) and S.R. (soumya@broadinstitute.org)

Supplementary Note 1
Supplementary Figures 1-15
Supplementary Tables 1-11
Supplementary Methods
Supplementary References

Supplementary Note 1

Development of Symphony mapping metrics

Although Symphony inherently assumes that all query cell types are present in the reference, users may not always know whether their data contains novel (“unseen”) query cell types. To help identify these situations, Symphony provides two metrics that quantify how well query cells are represented by the reference: *per-cell* mapping metric and *per-cluster* mapping metric. Both metrics are based on the Mahalanobis distance, a multivariate distance metric which measures the distance from a point (vector in multidimensional space) to a distribution (**Methods**). The *per-cell* metric gives a value to each query cell, whereas the *per-cluster* metric gives a value to each (user-defined) query cluster. Because the metric measures distance, higher values indicate a greater difference between the query and reference and therefore a worse mapping. In order to handle a large range of potential query-to-reference dataset differences, we do not prescribe specific cutoff values to use in all situations. Rather, users can select a threshold above which to flag query cells/clusters warranting further investigation or removal from the mapping. We explored Symphony’s behavior when the query contains unseen cell types as well as the performance of the mapping metrics in the analyses below.

Mapping confidence vs. prediction confidence

As a point of clarification, we note that mapping confidence is separate but related to the concept of prediction confidence. Symphony’s prediction confidence score reflects certainty in the annotation transfer step when the reference is assumed to contain the query cell state. It assigns lower confidence to query cells that lie “on the border” between two reference states (**Methods**).

Testing mapping metrics in different missing cell type scenarios

We first tested the metrics using the fetal liver hematopoiesis dataset, in three increasingly difficult scenarios. In each scenario, we artificially remove cell type(s) from the reference dataset prior to reference building, then mapped a held-out query donor containing all 27 cell types, including the now “unseen” types. We assessed how well each mapping metric could distinguish the missing type, as defined by AUC (which measures the ability to rank cells according to their probability of class membership, here missing vs. present in the reference). In aggregate, these case study scenarios show that the Symphony mapping metrics can be extremely useful in identifying novel cell states. However, the metrics may lack sensitivity in detecting very fine-grained cell state missing in the reference. Symphony typically maps these query states to the most similar reference state.

Scenario 1: Reference missing non-immune cells

In the easiest scenario, the reference did not contain hepatocytes, fibroblasts, and endothelial cells (the non-immune cell types). After mapping the query containing all cell types (**Supplementary Fig. 9a**), we found that the unseen non-immune query cell types were clearly distinguishable as having worse per-cell and per-cluster mapping metrics compared to the cell types captured in the reference (per-cell AUC = 0.997 and per-cluster AUC = 1.0) (**Supplementary Fig. 9b-d**).

Scenario 2: Reference missing myeloid cells

In a more difficult example, we built a reference missing a subset of immune cells: all cells of the myeloid lineage. Upon mapping the query (**Supplementary Fig. 10a**), we found that the distance metrics for the unseen myeloid cell types are generally higher than for the seen cells (per-cell AUC = 0.996, per-cluster AUC = 0.996) (**Supplementary Fig. 10b-d**). However, the distinguishability was somewhat lower compared to the first scenario, since the missing cell types are biologically more similar to the cell types in the reference. The distinguishability also varied by cell type along the myeloid lineage, where more differentiated myeloid cells (Kupffer cells and Mono-Mac) had the highest per-cell metrics (worst mapping). For unseen cell types that had the lowest metrics (better mapping), we found that they mapped onto biologically similar cell states in the reference. For example, the neutrophil-myeloid progenitor cells mapped onto reference hematopoietic stem cells (**Supplementary Fig. 10a**), which likely reflects their similar, less differentiated state. VCAM1+ erythroblastic island macrophages (VCAM1+ EI Macro.) cells are transcriptionally similar to both macrophages and erythroid cells[1]; supporting their mapping onto reference erythroid cells (**Supplementary Fig. 10a**).

Scenario 3: Reference missing Kupffer cells

In the most difficult scenario, we built a reference missing Kupffer cells, which are liver tissue-resident macrophages. This scenario is especially difficult because the reference contains biologically similar macrophage and monocyte states. In this case, Symphony maps the unseen query Kupffer cells onto their immediate precursor (Monocyte-Macrophage) state in the reference (**Supplementary Fig. 11a**). The mapping metrics are not able to clearly distinguish the Kupffer cells as novel (per-cell AUC = 0.633, per-cluster AUC = 0.963) (**Supplementary Fig. 11b-d**).

Comparison of Symphony mapping metrics to Seurat mapping score

Next, we sought to systematically compare the performance of the Symphony mapping metrics to the Seurat mapping score, using the 10x PBMCs dataset described previously. Using reference datasets (5' and 3'v2), we iteratively removed one broad cell type from the reference prior to reference building,

representing 7 different “missing cell type” scenarios: B, DC, HSC, Mono, MK, NK, and T. We built references using Symphony and Seurat for each scenario, mapped the query (3’v1) containing all cell types onto each reference, and then calculated the Symphony per-cell metric, Symphony per-cluster metric, and Seurat mapping score for each scenario (**Supplementary Fig. 12**).

When each method was permitted to select a unique cutoff value for each scenario to flag unseen cells, all three metrics performed comparably well (Symphony mean per-cell AUC = 0.88, per-cluster AUC = 0.86, Seurat AUC = 0.86; **Supplementary Fig. 13a**). Consistent with our observations in the fetal liver scenarios, the ability for mapping scores to detect novel populations highly depends on the identity of the missing cell type (**Supplementary Fig. 13a**). For example, it is easier for all three methods to call out missing B or T cells as novel than it is to identify NK cells or MKs as novel. We next calculated the AUCs for each method by aggregating all cells from all 7 scenarios together and using “seen” vs. “unseen” as the label to predict for each cell. When methods were made to choose the same cutoff values across all 7 scenarios, the AUCs are also highly similar across the three metrics (Symphony per-cell AUC = 0.926, Symphony per-cluster AUC = 0.994, Seurat mapping score AUC = 0.961; **Supplementary Fig. 13b**).

Supplementary Figures 1-15

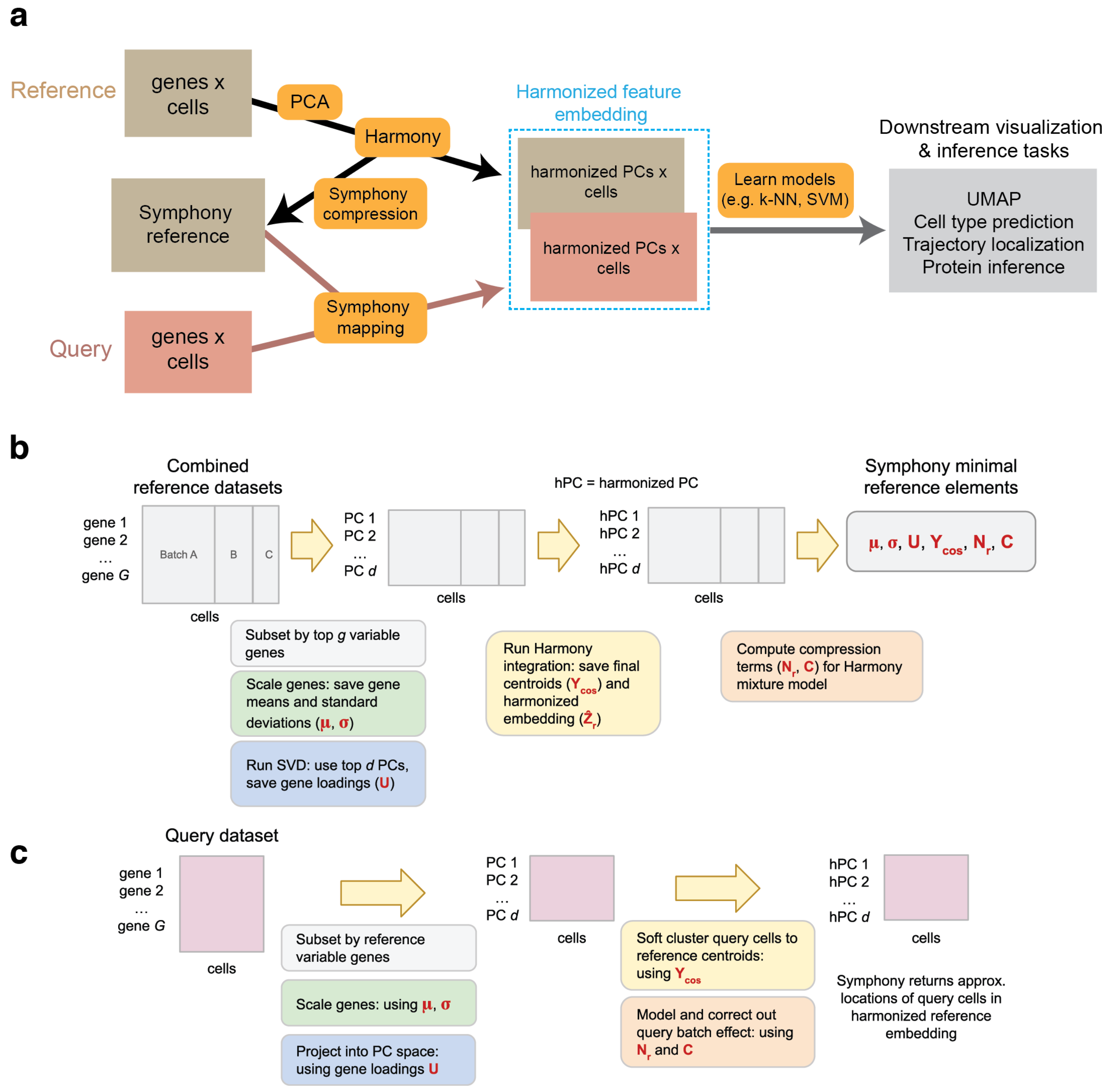

Supplementary Figure 1: Overview of reference mapping pipeline and Symphony data structures. (a) The overall analysis pipeline comprises various functions (orange boxes) that each perform a transformation on the data. Symphony mapping takes in a query gene expression matrix and a Symphony reference built from integrated reference datasets, and outputs the query cell locations in the harmonized feature embedding. Models trained on the reference feature embedding (e.g. cell type classifier) can transfer annotations to the query for various downstream tasks. **(b)** Steps of reference building algorithm. Reference datasets spanning multiple batches are aggregated into a single expression matrix on which PCA and Harmony integration is performed. The output of reference compression is the Symphony minimal reference elements, consisting of data structures $\boldsymbol{\mu}$, $\boldsymbol{\sigma}$, $\mathbf{U}$, $\mathbf{Y_{cos}}$, $\mathbf{N}_{\boldsymbol{r}}$, and $\mathbf{C}$ (red symbols, defined in figure itself and **Methods**). $\hat{\mathbf{Z}}_{\mathbf{r}}$ (the harmonized reference embedding) is not directly used for the mapping calculation but is saved for downstream annotation transfer. **(c)** Steps of query mapping algorithm, indicating where each reference element is used. Query cells are projected into reference PCA space, clustered to reference centroids, and corrected to harmonized space by removing query batch effects. Background colors for each outlined step in **(b)** and **(c)** delineate where the components calculated in reference building **(b)** are used in mapping **(c)**. “hPC” denotes harmonized PC.

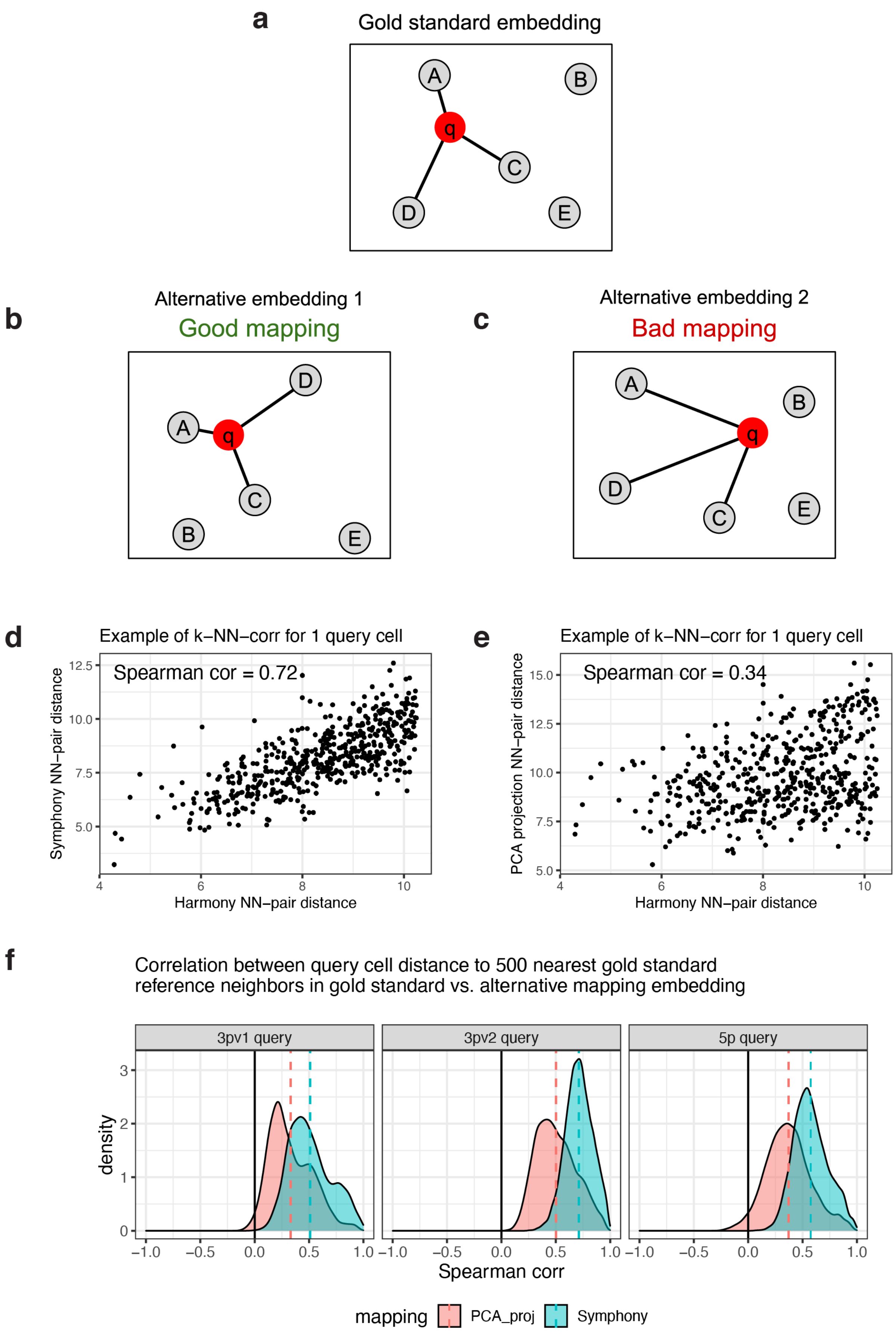
a
Gold standard embedding
A
B
q
C
D
E
b
Alternative embedding 1
Good mapping
c
Alternative embedding 2
Bad mapping
d
Example of k−NN−corr for 1 query cell
Spearman cor = 0.72
Symphony NN−pair distance
Harmony NN−pair distance
e
Example of k−NN−corr for 1 query cell
Spearman cor = 0.34
PCA projection NN−pair distance
Harmony NN−pair distance
f
Correlation between query cell distance to 500 nearest gold standard reference neighbors in gold standard vs. alternative mapping embedding
3pv1 query
3pv2 query
5p query
density
Spearman corr
mapping
PCA_proj
Symphony

Supplementary Figure 2: Nearest neighbor correlation (k-NN-corr) metric. The k-NN-correlation metric assesses how well an alternative embedding recapitulates the structure of a gold standard embedding. k-NN-corr is asymmetric in that it matters which of the two embeddings is selected as the gold standard. Consider a gold standard embedding **(a)** and two alternative embeddings **(b)** and **(c)**, representing a good mapping and a bad mapping, respectively. For a given query cell q (red circle), we identify its top k nearest reference cell neighbors (gray circles) in the gold standard embedding (k = 3 depicted by black edges) and calculate the distance between the query cell and each neighbor. The distances between the same query-reference neighbor pairs are then calculated in the alternate embedding. k-NN-corr is the Spearman correlation between the distances in the gold standard vs. alternative embedding, ranging from -1 to +1. Example k-NN-corr for one query cell and k = 500 for the **(d)** Symphony embedding and **(e)** PCA projection embedding. **(f)** k-NN-corr distribution across query cells for k = 500 and a gold standard Harmony embedding, for either the Symphony embeddings (blue) or a simple PCA projection with no correction step (light red), faceted by query dataset. Dotted vertical lines denote mean k-NN-corr for a given query and mapping method.

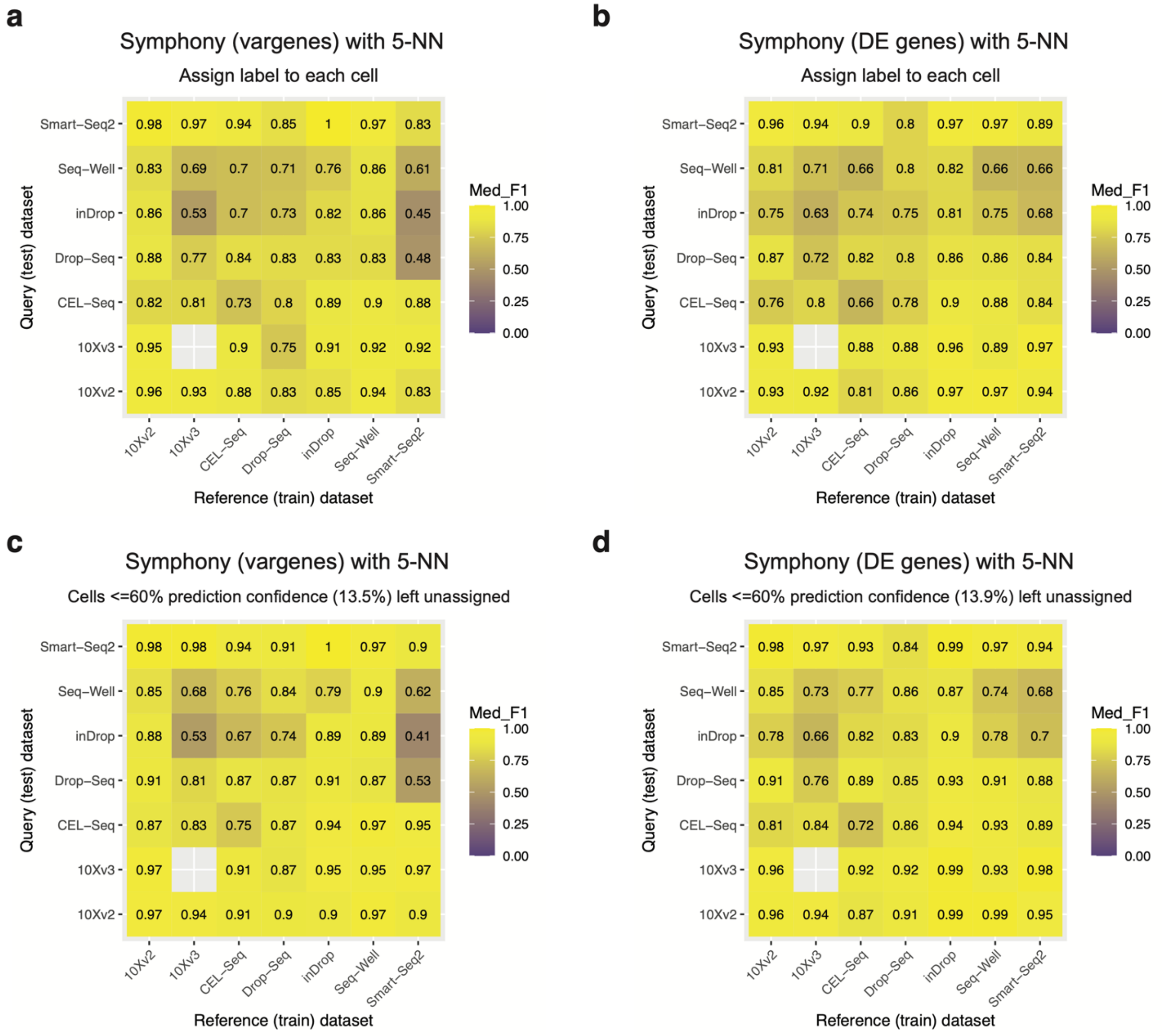

Supplementary Figure 3: Symphony performance on PbmcBench benchmark. Following the cross-technology PBMC benchmarking experiment from Abdelaal et al. (2019)[2], we ran a total of 48 train-test experiments per Symphony-based classifier. Two different versions of the Symphony feature embeddings were generated depending on variable gene selection method: top 2,000 variable genes (vargenes) or top 20 differentially genes (DEGs) expressed per cell type. Symphony embeddings were used to train 3 downstream classifiers: k-NN (k = 5), SVM with radial kernel, and multinomial logistic regression with ridge. **(a, b)** Median cell type F1-score across 48 experiments for the 5-NN classifier with **(a)** variable gene selection and **(b)** DEG selection, assigning a label to every query cell. Non-diagonal values represent train on one technology, test on another (42 experiments, all with donor 1). Values along the diagonal indicate train on donor 1, test on donor 2 of the same technology (6 experiments; missing square because donor 2 not sequenced with 10x v3). **(c, d)** is analogous to **(a, b)** except considering only “high-confidence” cells (predicted with >60% confidence, i.e. ≥4 reference neighbors with winning vote) for F1 score calculations. Colored by median F1 score.

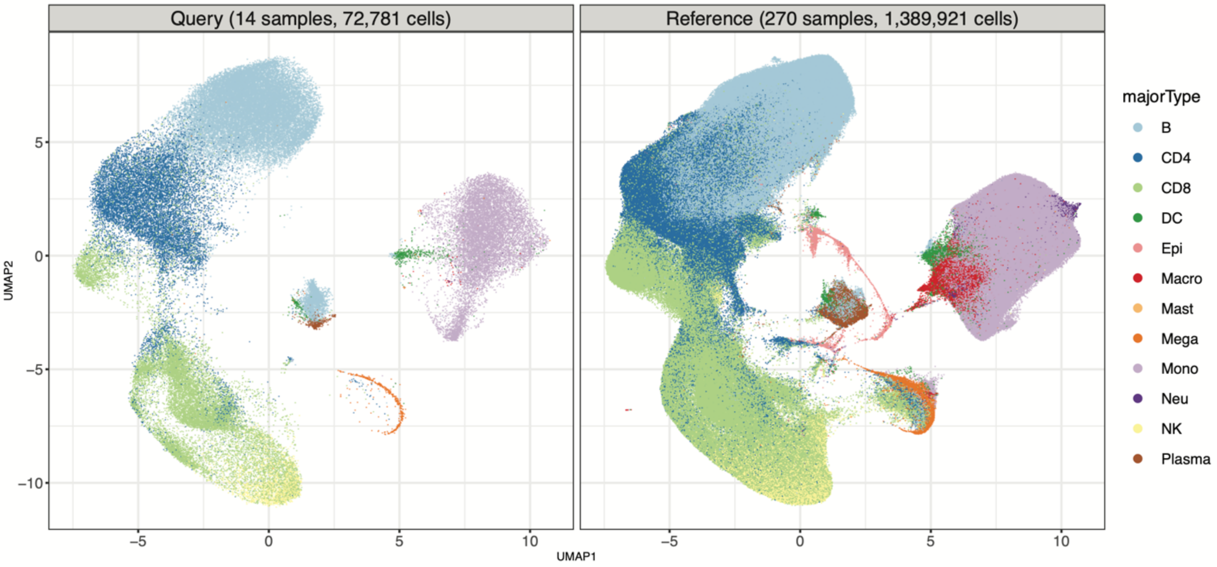

Supplementary Figure 4: Symphony constructs and maps to a multi-million cell atlas. To demonstrate Symphony's scalability to multi-million cell atlases, we used a large-scale scRNA-seq dataset (Ren et al., 2021)[3]. We built a Symphony reference of 1.39 million cells from 270 samples and mapped a held-out set of 14 samples (n = 72,781 cells) as the query. UMAP plots show the resulting embeddings of reference and query cells, colored by author-defined major cell type.

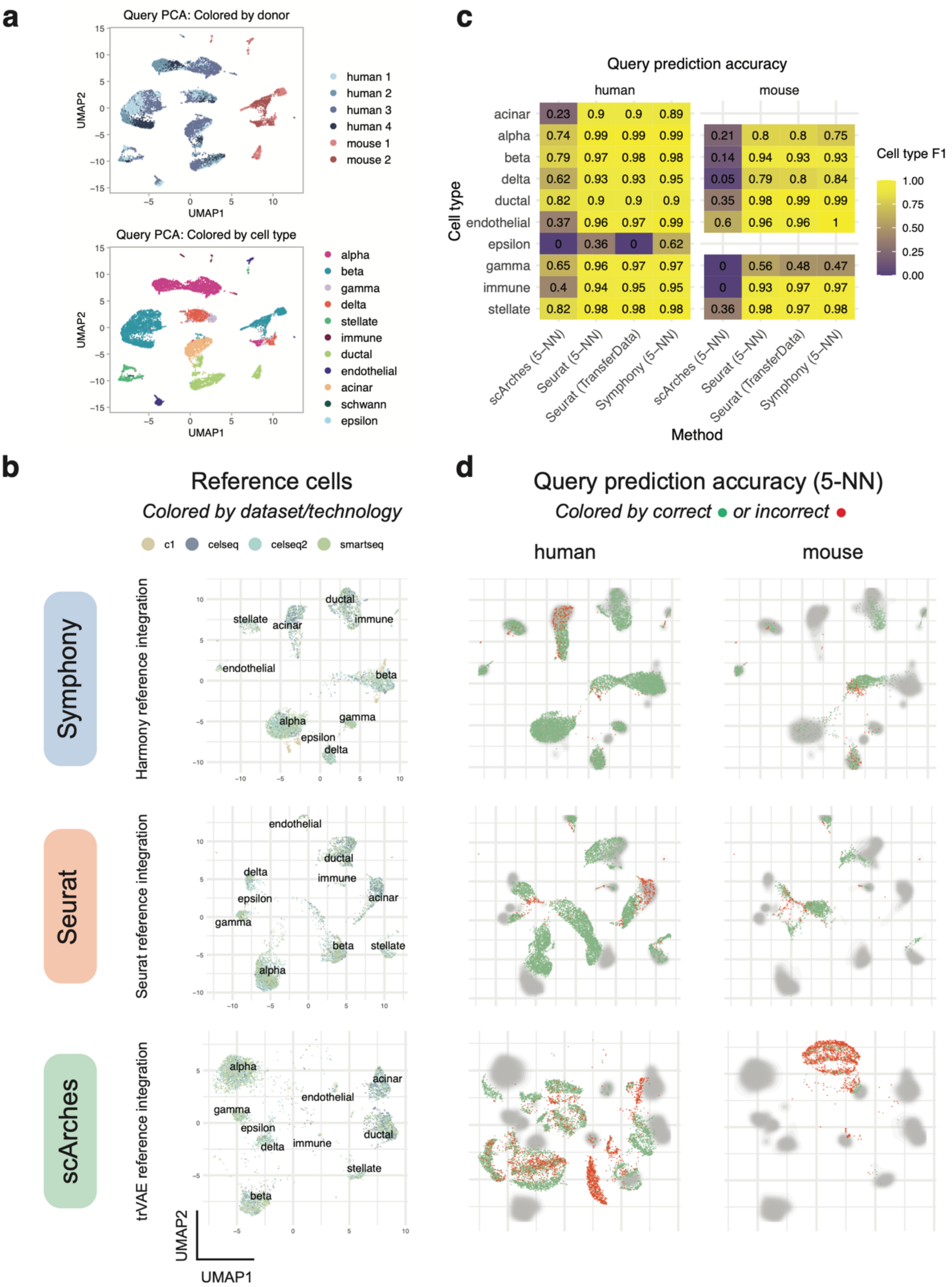
a
Query PCA: Colored by donor
human 1
human 2
human 3
human 4
mouse 1
mouse 2
UMAP2
UMAP1
Query PCA: Colored by cell type
alpha
beta
gamma
delta
stellate
immune
ductal
endothelial
acinar
schwann
epsilon
c
Query prediction accuracy
human
mouse
Cell type
acinar 0.23 0.9 0.9 0.89
alpha 0.74 0.99 0.99 0.99 0.21 0.8 0.8 0.75
beta 0.79 0.97 0.98 0.98 0.14 0.94 0.93 0.93
delta 0.62 0.93 0.93 0.95 0.05 0.79 0.8 0.84
ductal 0.82 0.9 0.9 0.9 0.35 0.98 0.99 0.99
endothelial 0.37 0.96 0.97 0.99 0.6 0.96 0.96 1
epsilon 0 0.36 0 0.62
gamma 0.65 0.96 0.97 0.97 0 0.56 0.48 0.47
immune 0.4 0.94 0.95 0.95 0 0.93 0.97 0.97
stellate 0.82 0.98 0.98 0.98 0.36 0.98 0.97 0.98
Cell type F1
1.00
0.75
0.50
0.25
0.00
scArches (5–NN)
Seurat (5–NN)
Seurat (TransferData)
Symphony (5–NN)
Method
b
Reference cells
Colored by dataset/technology
c1
celseq
celseq2
smartseq
Symphony
Harmony reference integration
Seurat
Seurat reference integration
scArches
trVAE reference integration
UMAP2
UMAP1
d
Query prediction accuracy (5-NN)
Colored by correct or incorrect
human
mouse

Supplementary Figure 5: Comparison of Symphony to alternative reference mapping methods on a cross-species pancreas benchmark. **(a)** Standard PCA pipeline applied to the Baron et al.[4] query dataset exhibits strong species and donor effects, demonstrating the need for within-query integration. We benchmarked Symphony mapping (on a Harmony-integrated reference), Seurat mapping (on a Seurat anchor-based-integrated reference), and scArches mapping (on a trVAE-integrated reference). For each approach, we built an integrated reference **(b)**, mapped the query, then predicted query cell types using a 5-NN classifier to transfer annotations using the respective reference embedding. For Seurat, we also tested the TransferData function. **(c)** Query cell prediction accuracy by species for each method as measured by cell type F1-score (color), with author-defined ground truth labels. Mouse samples did not have any acinar or epsilon cells. The resulting joint cell embedding for each tool was visualized by UMAP **(b, d)**: **(b)** reference cells colored by dataset/technology, **(d)** query cells colored by correct (green) or incorrect (red) cell type prediction for 5-NN classifiers.

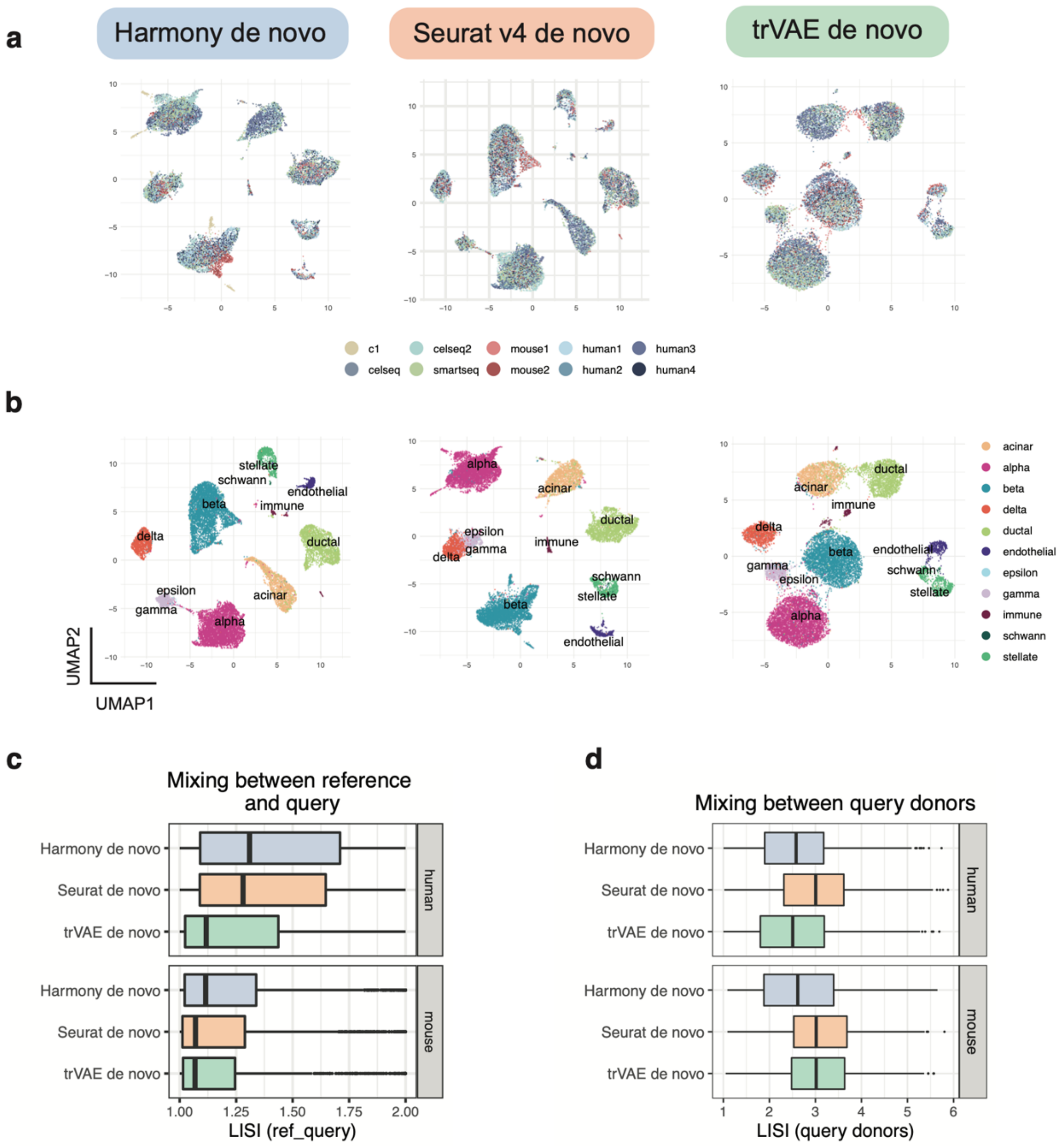

Supplementary Figure 6: Comparison of de novo integration methods for harmonizing all five pancreatic islet cell datasets. As a comparison to reference mapping (**Fig. 4**), we integrated all five pancreatic islet cell technologies (n = 16,342 cells) using three *de novo* integration methods: Harmony, Seurat anchor-based integration, and trVAE. UMAP visualizations for the integrated embedding colored by batch **(a)** and cell types **(b)** for each method. Cell types for reference datasets (c1, celseq, celseq2, smartseq) were defined within each dataset separately based on marker genes. Query cell types were defined by Baron et al. **(c, d)** Degree of mixing between reference and query datasets **(c)** and mixing between query donors **(d)** was measured with LISI metric on query cell neighborhoods (human: n = 8,569 cells from 4 donors, mouse: n = 1,866 cells from 2 donors) for each method, demonstrating comparable mixing among *de novo* integration methods (compare to **Fig. 4e-f**). Boxplot center line represents the median; lower and upper box limits represent the 25% and 75% quantiles, respectively; whiskers extend to box limit ±1.5 × IQR; outlying points plotted individually.

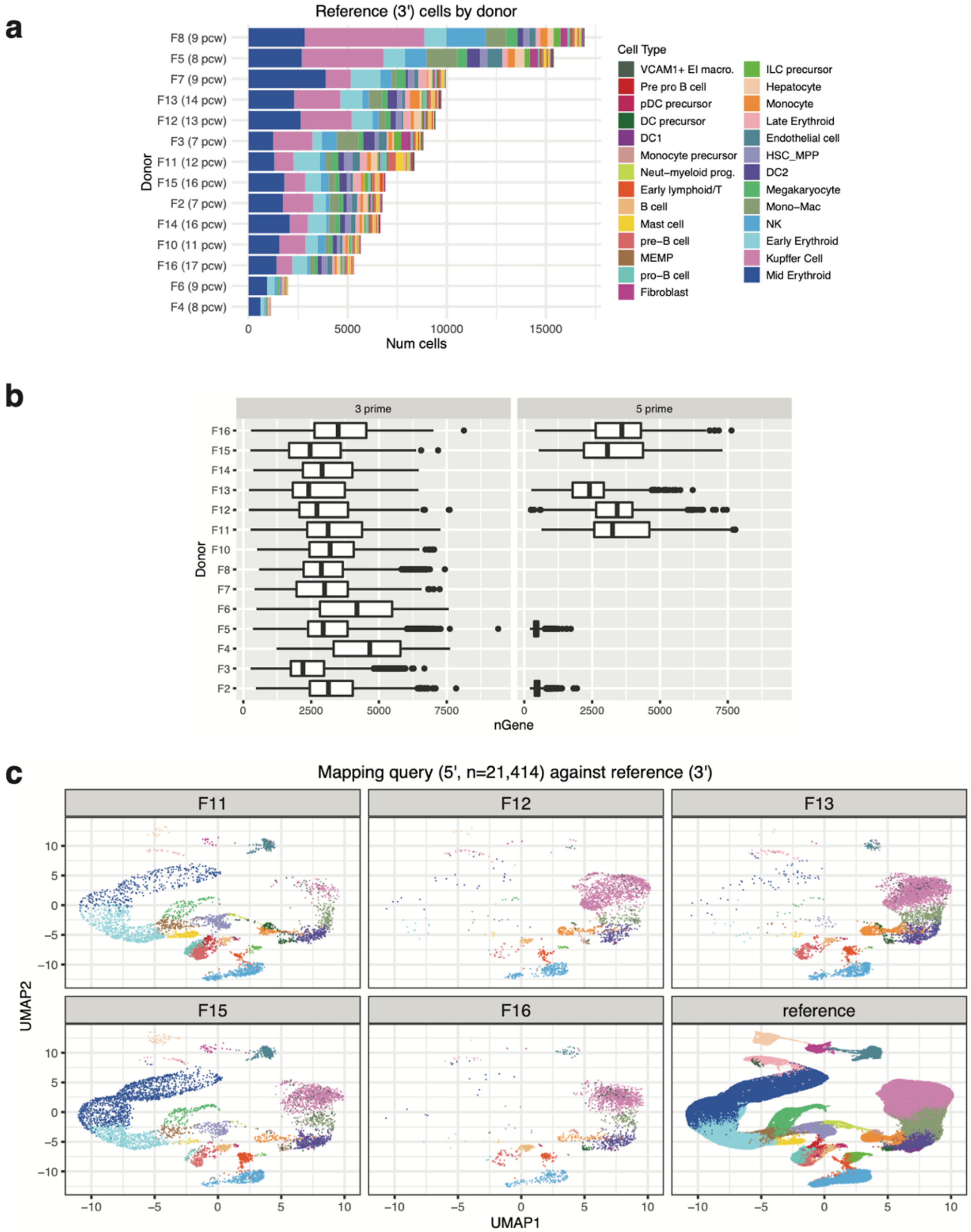

Supplementary Figure 7: Mapping to a fetal liver hematopoiesis trajectory. (a) Size and cell type (color) composition of each donor sample in the 10x 3' reference dataset across 27 author-defined cell types from Popescu et al. (2019). pcw = post-conception weeks. **(b)** Library complexity in number of genes (nGene) for each sample in reference (10x 3') and query (10x 5') datasets, showing low complexity for donor F2 and F5 for 5'-sequenced samples (n = 3,953 cells, removed from further analysis). Boxplot center line represents the median; lower and upper box limits represent the 25% and 75% quantiles, respectively; whiskers extend to box limit ±1.5 × IQR; outlying points plotted individually. **(c)** UMAP projections of query cells into reference UMAP space after Symphony mapping, faceted by query donor, colored by cell type. Reference UMAP embedding in bottom-right.

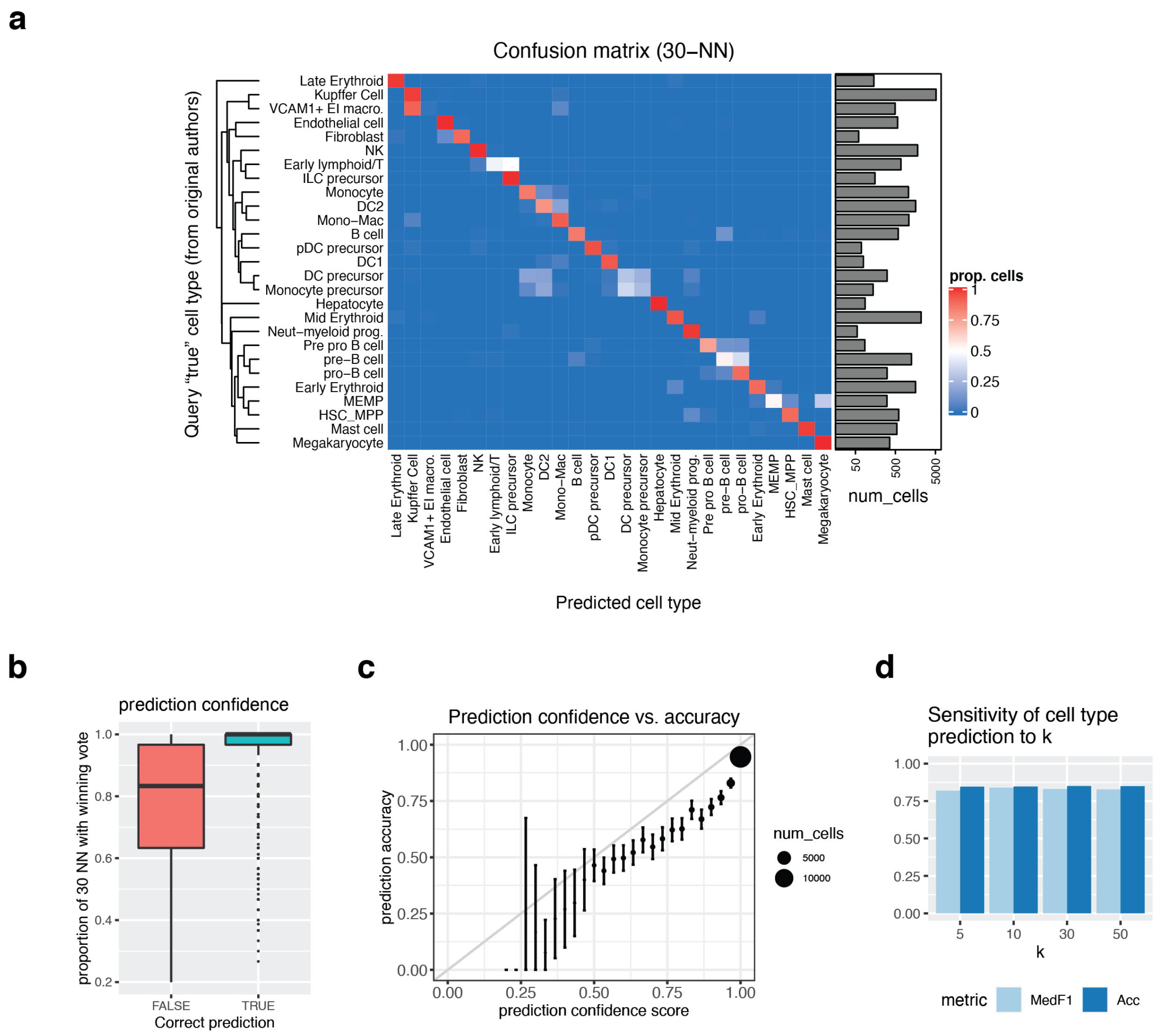

Supplementary Figure 8: Fetal liver hematopoiesis cell type classification. We mapped the query (5', n = 21,414 cells, 5 donors) dataset onto the reference (3', n = 113,063 cells, 14 donors) and assessed cell type classification accuracy across 27 fine-grained cell types: **(a)** Cell type confusion matrix for 30-NN cell type classification, colored by the proportion of query cells in a given true cell type that was classified to each reference label (rows sum to 1). True cell type is defined by the original authors (Popescu et al., 2019)[1]. Rows (true query cell types) are sorted by hierarchical clustering on the average gene expression (all genes) for the cell types to order similar types together. Bar graph (right) shows population size for each cell type. **(b)** Boxplots showing prediction confidence (measured as proportion of nearest reference neighbors with winning vote) across query cells for 30-NN, colored by whether the cell received a correct (n = 18,195 cells) vs. incorrect (n = 3,219 cells) prediction. Boxplot center line represents the median; lower and upper box limits represent the 25% and 75% quantiles, respectively; whiskers extend to box limit ±1.5 × IQR; outlying points plotted individually. **(c)** Relationship between prediction confidence score (x-axis; proportion of 30-NN with winning vote) and prediction accuracy (y-axis; proportion of correctly classified cells), across all n = 21,414 query cells, showing that the two measures track closely. Point size is the number of cells with a given prediction confidence score. Error bars show 95% C.I. using the binomial proportion confidence interval, centered at the mean. **(d)** Median cell type F1 and overall classification accuracy across varying values of k = 5, 10, 30, 50 used for query cell type prediction.

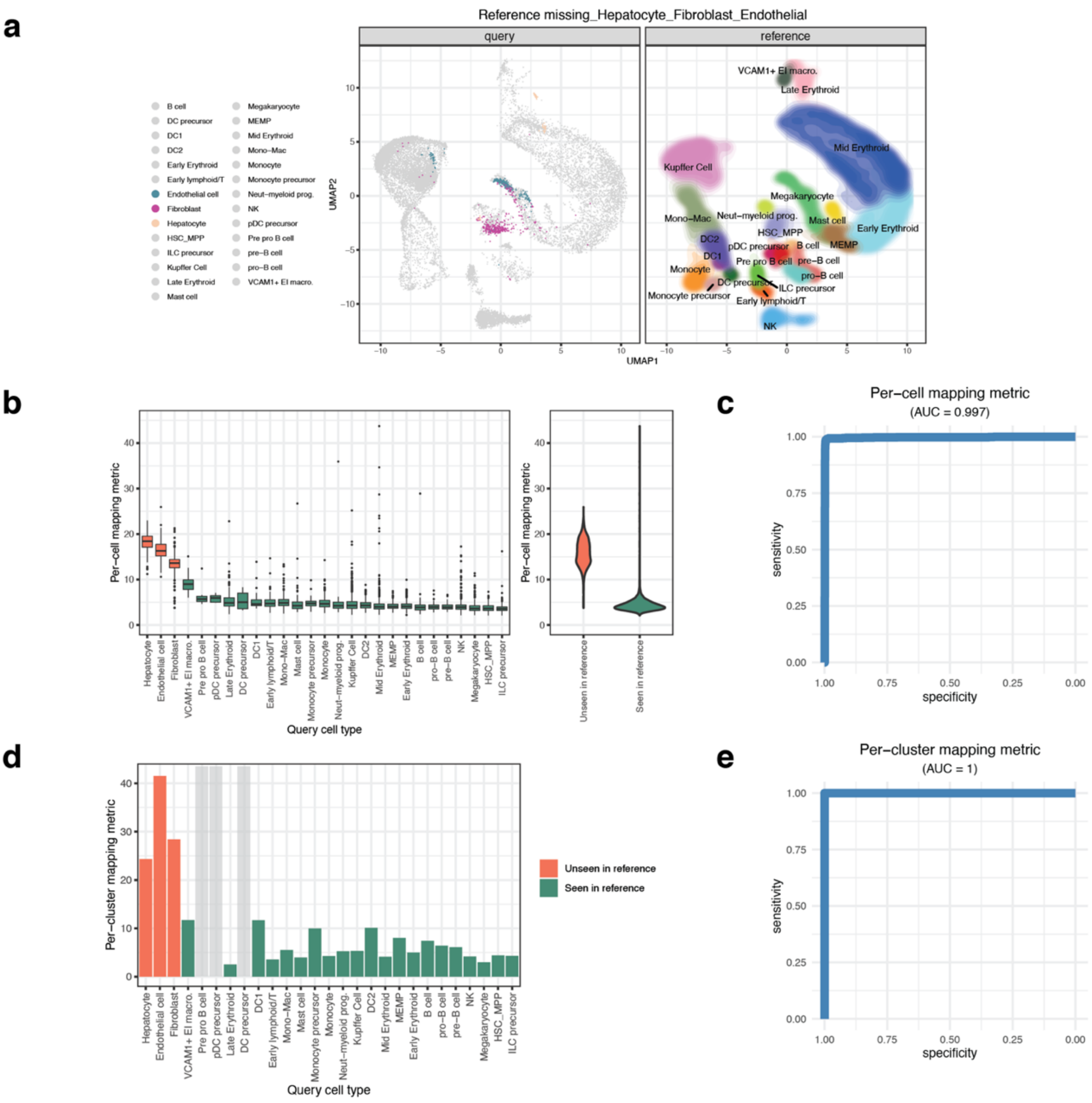
a
Reference missing_Hepatocyte_Fibroblast_Endothelial
query
reference
B cell
DC precursor
DC1
DC2
Early Erythroid
Early lymphoid/T
Endothelial cell
Fibroblast
Hepatocyte
HSC_MPP
ILC precursor
Kupffer Cell
Late Erythroid
Mast cell
Megakaryocyte
MEMP
Mid Erythroid
Mono−Mac
Monocyte
Monocyte precursor
Neut−myeloid prog.
NK
pDC precursor
Pre pro B cell
pre−B cell
pro−B cell
VCAM1+ EI macro.
UMAP1
UMAP2
b
Per−cell mapping metric
Query cell type
Unseen in reference
Seen in reference
c
Per−cell mapping metric
(AUC = 0.997)
sensitivity
specificity
d
Per−cluster mapping metric
Query cell type
Unseen in reference
Seen in reference
e
Per−cluster mapping metric
(AUC = 1)
sensitivity
specificity

Supplementary Figure 9: Scenario where reference is missing non-immune cells. (a) UMAP of harmonized embedding, with reference (n = 89,566) shown as density colored by cell type and query cells (n = 16,945) plotted with unseen states colored (and present states in gray), to highlight where the unseen cells map to. **(b)** Symphony *per-cell* mapping metrics calculated on the query cells, colored by whether cell types are unseen vs. seen, plotted by individual cell types as a boxplot (left, in descending order by mean) or aggregating all the unseen vs. seen cell types together in a violin plot (right). Unseen query cell types: Endothelial cells (n = 321 cells), Fibroblasts (n = 361), Hepatocytes (n = 306). Seen query cell types (n cells): B cell (87), DC precursor (14), DC1 (56), DC2 (292), Early Erythroid (1,131), Early lymphoid/T (57), HSC/MPP (292), ILC precursor (340), Kupffer Cell (6,022), Late Erythroid (235), Mast cell (78), Megakaryocyte (570), MEMP (166), Mid Erythroid (2,833), Mono-Mac (1,035), Monocyte (375), Monocyte precursor (44), Neut.-myeloid progenitor (91), NK (1,976), pDC precursor (9), Pre pro B cell (12), pre-B cell (84), pro-B cell (106), VCAM1+ Erythroblastic Island macrophage (52). Boxplot center line represents the median; lower and upper box limits represent the 25% and 75% quantiles, respectively; whiskers extend to box limit ±1.5 × IQR; outlying points plotted individually. **(c)** AUC for the per-cell metric, measuring how distinguishable seen vs. unseen cells are. **(d)** Symphony *per-cluster* mapping metrics for each query cell type, with x-axis ordered the same as in **(b)**, colored by unseen vs. seen. Light gray shading indicates clusters too small to calculate the metric (n < 2 × dimensionality, **Methods**). **(e)** AUC for per-cluster metric across all query cells (all cells of the same cluster receive the same metric).

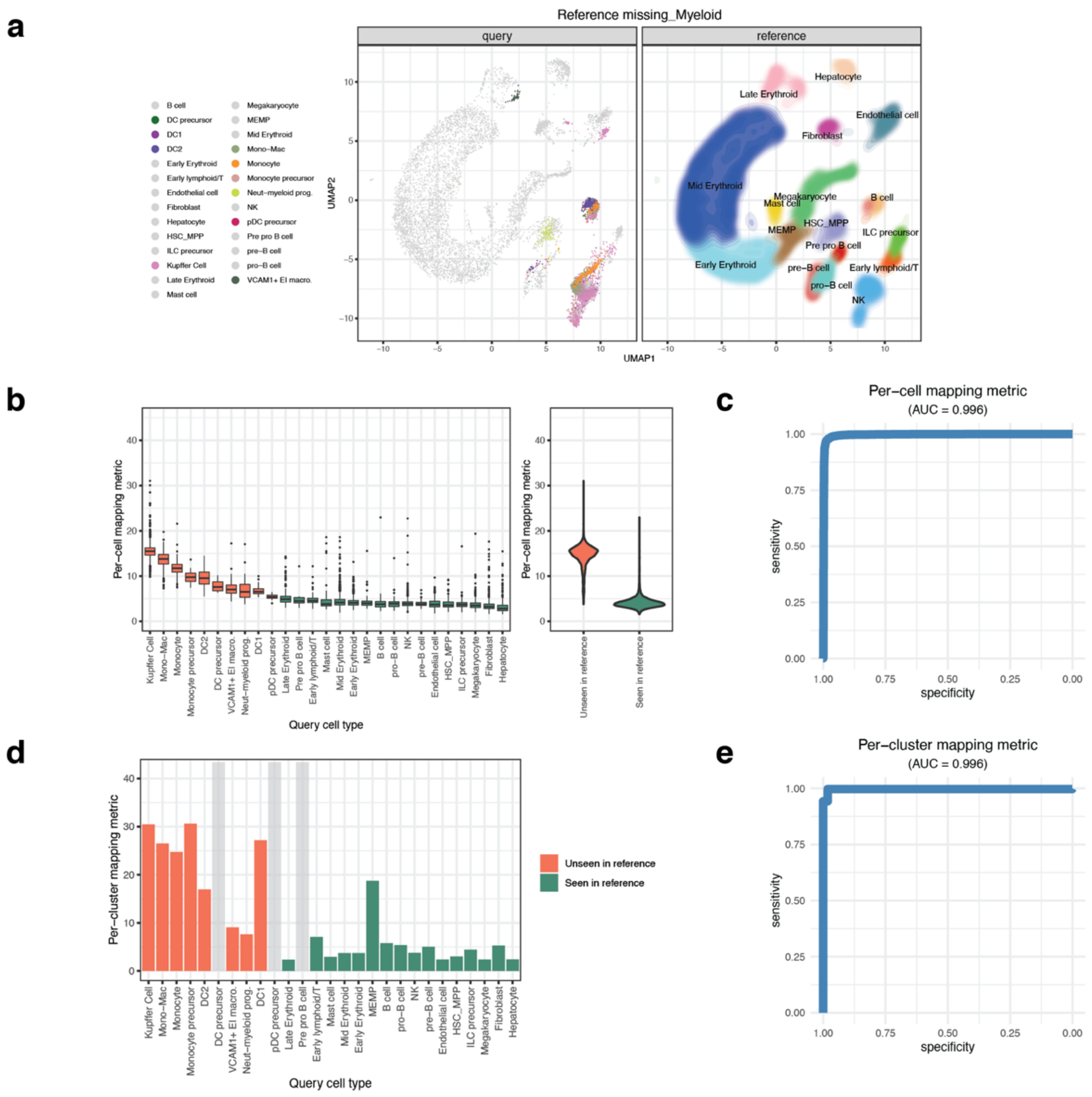
a
Reference missing_Myeloid
query
reference
UMAP1
UMAP2
B cell
DC precursor
DC1
DC2
Early Erythroid
Early lymphoid/T
Endothelial cell
Fibroblast
Hepatocyte
HSC_MPP
ILC precursor
Kupffer Cell
Late Erythroid
Mast cell
Megakaryocyte
MEMP
Mid Erythroid
Mono−Mac
Monocyte
Monocyte precursor
Neut−myeloid prog.
NK
pDC precursor
Pre pro B cell
pre−B cell
pro−B cell
VCAM1+ EI macro.
b
Per−cell mapping metric
Query cell type
Unseen in reference
Seen in reference
c
Per−cell mapping metric
(AUC = 0.996)
sensitivity
specificity
d
Per−cluster mapping metric
Query cell type
Unseen in reference
Seen in reference
e
Per−cluster mapping metric
(AUC = 0.996)
sensitivity
specificity

Supplementary Figure 10: Scenario where reference is missing myeloid lineage cells. (a) UMAP of harmonized embedding, with reference (n = 64,049) shown as density colored by cell type and query cells (n = 16,945) plotted with unseen states colored (and present states in gray), to highlight where the unseen cells map to. **(b)** Symphony *per-cell* mapping metrics calculated on the query cells, colored by whether cell types are unseen vs. seen, plotted by individual cell types as a boxplot (left, in descending order by mean) or aggregating all the unseen vs. seen cell types together in a violin plot (right). Unseen query cell types: Kupffer cells (n = 6,022 cells), Mono-Mac (n = 1,035), Monocyte (n = 375), Monocyte precursor (n = 44), DC1 (n = 56), DC2 (n = 292), VCAM1+ Erythroblastic Island macrophage (n = 52), Neut.-myeloid progenitor (n = 91), DC precursor (n = 14), pDC precursor (n = 9). Seen query cell types (n cells): B cell (87), Early Erythroid (1,131), Early lymphoid/T (57), HSC/MPP (292), ILC precursor (340), Endothelial cells (321), Fibroblasts (361), Hepatocytes (306), Late Erythroid (235), Mast cell (78), Megakaryocyte (570), MEMP (166), Mid Erythroid (2,833), NK (1,976), Pre pro B cell (12), pre-B cell (84), pro-B cell (106). Boxplot center line represents the median; lower and upper box limits represent the 25% and 75% quantiles, respectively; whiskers extend to box limit ±1.5 × IQR; outlying points plotted individually. **(c)** AUC for the per-cell metric, measuring how distinguishable seen vs. unseen cells are. **(d)** Symphony *per-cluster* mapping metrics for each query cell type, with x-axis ordered the same as in **(b)**, colored by unseen vs. seen. Light gray shading indicates clusters too small to calculate the metric (n < 2 × dimensionality, **Methods**). **(e)** AUC for per-cluster metric across all query cells (all cells of the same cluster receive the same metric).

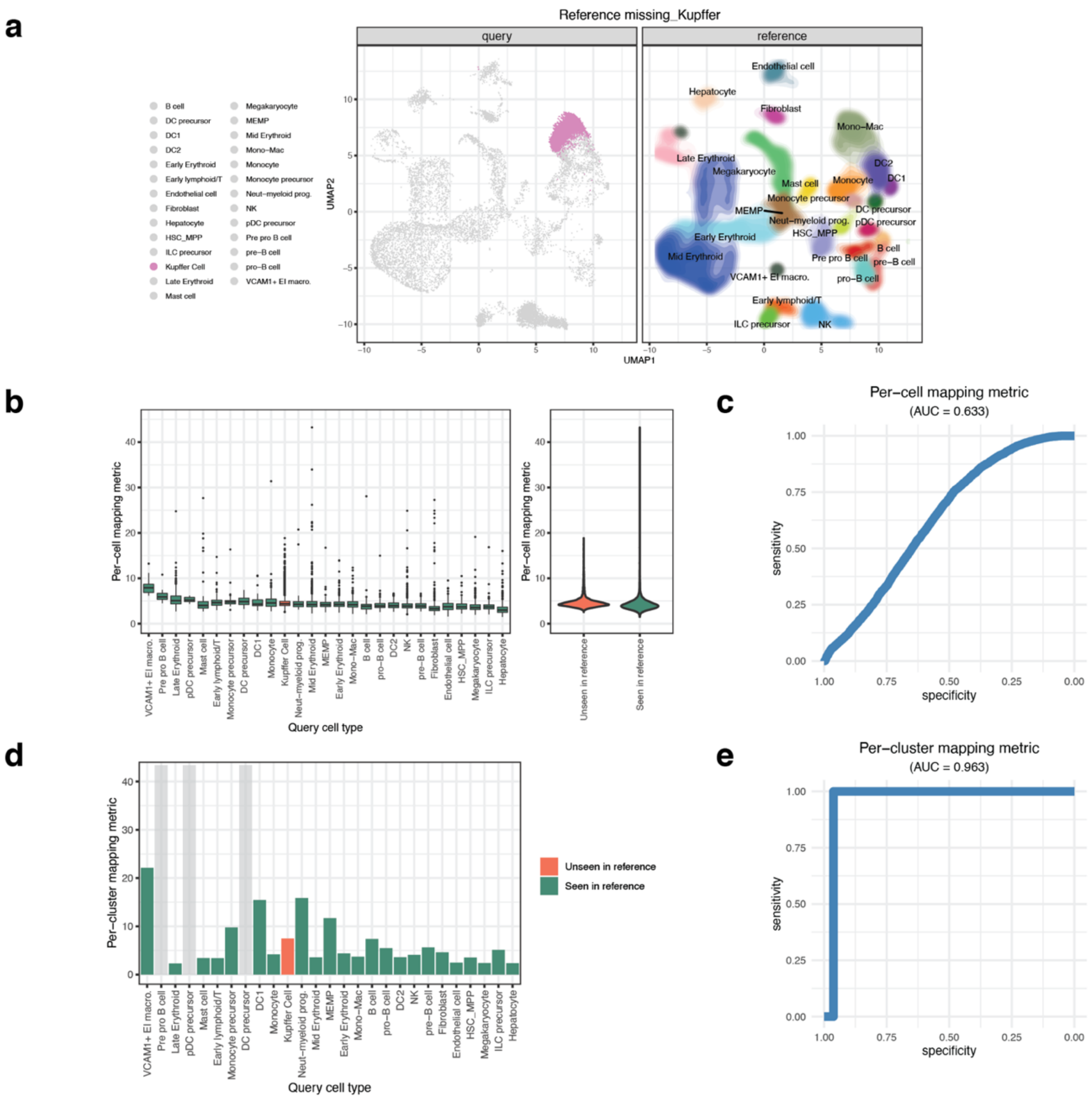

a
Reference missing_Kupffer
query
reference
UMAP1
UMAP2
B cell
DC precursor
DC1
DC2
Early Erythroid
Early lymphoid/T
Endothelial cell
Fibroblast
Hepatocyte
HSC_MPP
ILC precursor
Kupffer Cell
Late Erythroid
Mast cell
Megakaryocyte
MEMP
Mid Erythroid
Mono−Mac
Monocyte
Monocyte precursor
Neut−myeloid prog.
NK
pDC precursor
Pre pro B cell
pre−B cell
pro−B cell
VCAM1+ EI macro.
b
Per−cell mapping metric
Query cell type
Unseen in reference
Seen in reference
c
Per−cell mapping metric
(AUC = 0.633)
sensitivity
specificity
d
Per−cluster mapping metric
Query cell type
Unseen in reference
Seen in reference
e
Per−cluster mapping metric
(AUC = 0.963)
sensitivity
specificity

Supplementary Figure 11: Scenario where reference is missing Kupffer cells. (a) UMAP of harmonized embedding, with reference (n = 77,299) shown as density colored by cell type and query cells (n = 16,945) plotted with unseen states colored (and present states in gray), to highlight where the unseen cells map to. **(b)** Symphony *per-cell* mapping metrics calculated on the query cells, colored by whether cell types are unseen vs. seen, plotted by individual cell types as a boxplot (left, in descending order by mean) or aggregating all the unseen vs. seen cell types together in a violin plot (right). Unseen query cell type: Kupffer cells (n = 6,022 cells). Seen query cell types (n cells): B cell (87), DC precursor (14), DC1 (56), DC2 (292), Early Erythroid (1,131), Early lymphoid/T (57), HSC/MPP (292), ILC precursor (340), Endothelial cells (321 cells), Fibroblasts (361), Hepatocytes (306), Late Erythroid (235), Mast cell (78), Megakaryocyte (570), MEMP (166), Mid Erythroid (2,833), Mono-Mac (1,035), Monocyte (375), Monocyte precursor (44), Neut.-myeloid progenitor (91), NK (1,976), pDC precursor (9), Pre pro B cell (12), pre-B cell (84), pro-B cell (106), VCAM1+ Erythroblastic Island Macrophage (52). Boxplot center line represents the median; lower and upper box limits represent the 25% and 75% quantiles, respectively; whiskers extend to box limit ±1.5 × IQR; outlying points plotted individually. **(c)** AUC for the per-cell metric, measuring how distinguishable seen vs. unseen cells are. **(d)** Symphony *per-cluster* mapping metrics for each query cell type, with x-axis ordered the same as in **(b)**, colored by unseen vs. seen. Light gray shading indicates clusters too small to calculate the metric (n < 2 × dimensionality, **Methods**). **(e)** AUC for per-cluster metric across all query cells (all cells of the same cluster receive the same metric).

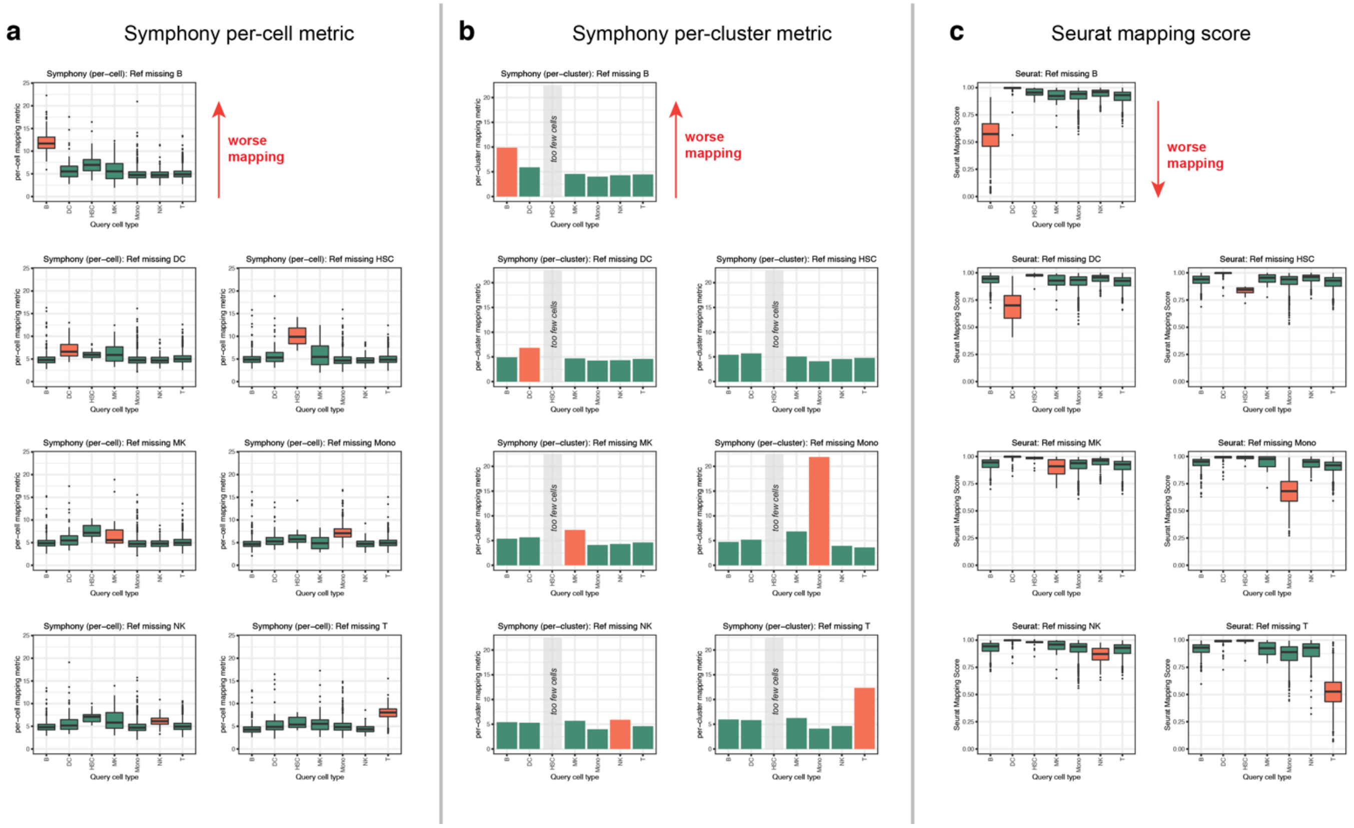

Supplementary Figure 12: Symphony mapping metrics and Seurat mapping score across PBMCs missing cell type scenarios. In a total of 7 “missing cell type” scenarios, we built references with datasets (3’v2 and 5’, total n = 15,813 cells) each with one major cell type artificially removed (B, DC, HSC, MK, Mono, NK, or T). **(a)** Onto each “missing cell type” reference, we mapped a separate query dataset (3’v1, n = 4,758 cells) containing all cell types: B (n = 589 cells), DC (n = 78), HSC (n = 21), MK (n = 30), Mono (n = 1,193), NK (n = 291), and T (n = 2,556). We calculated Symphony *per-cell* metrics for query cells across the scenarios (title of boxplot indicates the missing type). Query cells are grouped by cell type and colored by seen (green) vs. unseen (orange) in the reference for that scenario. Higher values indicate worse mapping. Boxplot center line represents the median; lower and upper box limits represent the 25% and 75% quantiles, respectively; whiskers extend to box limit ±1.5 × IQR; outlying points plotted individually. **(b)** Symphony *per-cluster* metrics for each scenario (1 value assigned to each query cluster), colored by seen (green) vs. unseen (orange). Higher values indicate worse mapping. Light gray “too few cells” bar indicates that the HSC cluster was too small (n = 21 cells) to calculate the per-cluster metric (**Methods**). **(c)** Seurat mapping confidence scores for the same scenarios with Seurat reference mapping pipeline. Lower values indicate worse mapping. Cell numbers and boxplot boundaries defined the same way as in **(a)**.

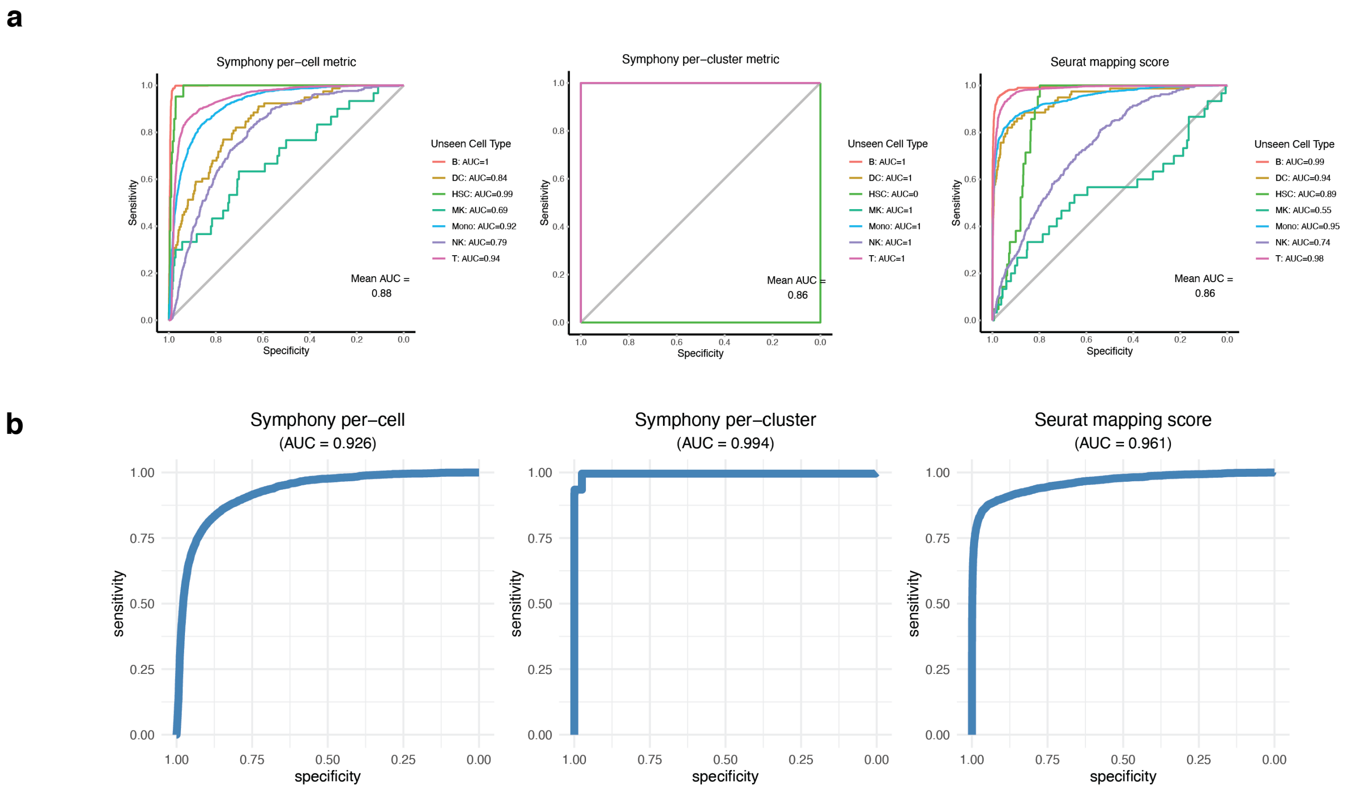

Supplementary Figure 13: ROC curves for Symphony metrics and Seurat mapping score across PBMCs missing cell type scenarios. AUCs were calculated across all query cells in each scenario using a binary label of missing vs. present in the reference as the ground truth for prediction. We generated ROCs for each metric in two ways: **(a)** considering each scenario separately (threshold values independent across scenarios) and **(b)** aggregating cells across all 7 scenarios together for a single calculation. For the Symphony per-cell metric and Seurat mapping score, each query cell is assigned its own value, whereas for the Symphony per-cluster metric, all cells from the same cluster are assigned the same value. The HSC cluster (n = 21 cells) was too small to calculate a per-cluster score and all HSCs were assigned a distance of 0 in all scenarios (unable to be flagged as novel) for inclusion in AUC calculations.

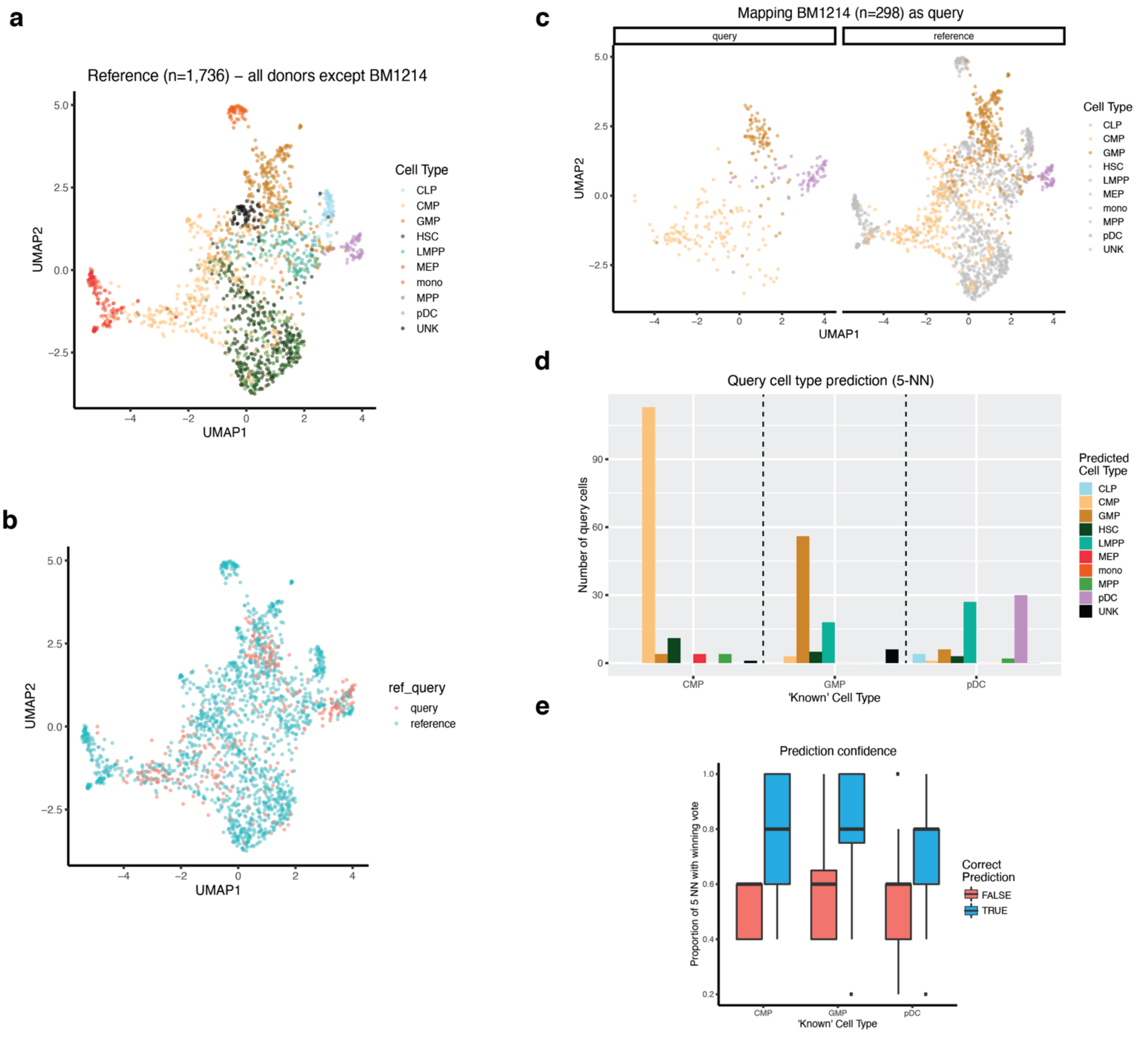

Supplementary Figure 14: Extending Symphony to scATAC-seq data. We built a reference using a scATAC-seq dataset (Buenrostro et al., 2018)[5], then mapped a held-out donor as the query. **(a)** Symphony reference embedding (n = 1,736) built from all donors except BM1214 (n = 298), colored by "known" cell type. UMAP shows regions of related cell types along Lymphoid, Myeloid, and Erythroid differentiation pathways as in Buenrostro et al. **(b, c)** Symphony mapping embedding, colored by **(b)** reference or query or **(c)** "known" cell type. **(d)** Barplot showing, for each of the 3 "known" cell types present in the query (CMP, GMP, and pDC), the number of query cells predicted across each of the cell types by Symphony (5-NN). **(e)** Prediction confidence scores for the query cells, measuring the proportion of 5 nearest reference neighbors supporting the predicted cell type label, colored by whether the query was ultimately predicted correctly (n = 113, 56, and 30 cells for CMP, GMP, and pDC, respectively) or incorrectly (n = 24, 32, and 43 cells for CMP, GMP, and pDC, respectively). Boxplot center line represents the median; lower and upper box limits represent the 25% and 75% quantiles, respectively; whiskers extend to box limit ±1.5 × IQR; outlying points plotted individually. Hematopoietic cell type abbreviations are as in Buenrostro et al.; UNK = unknown.

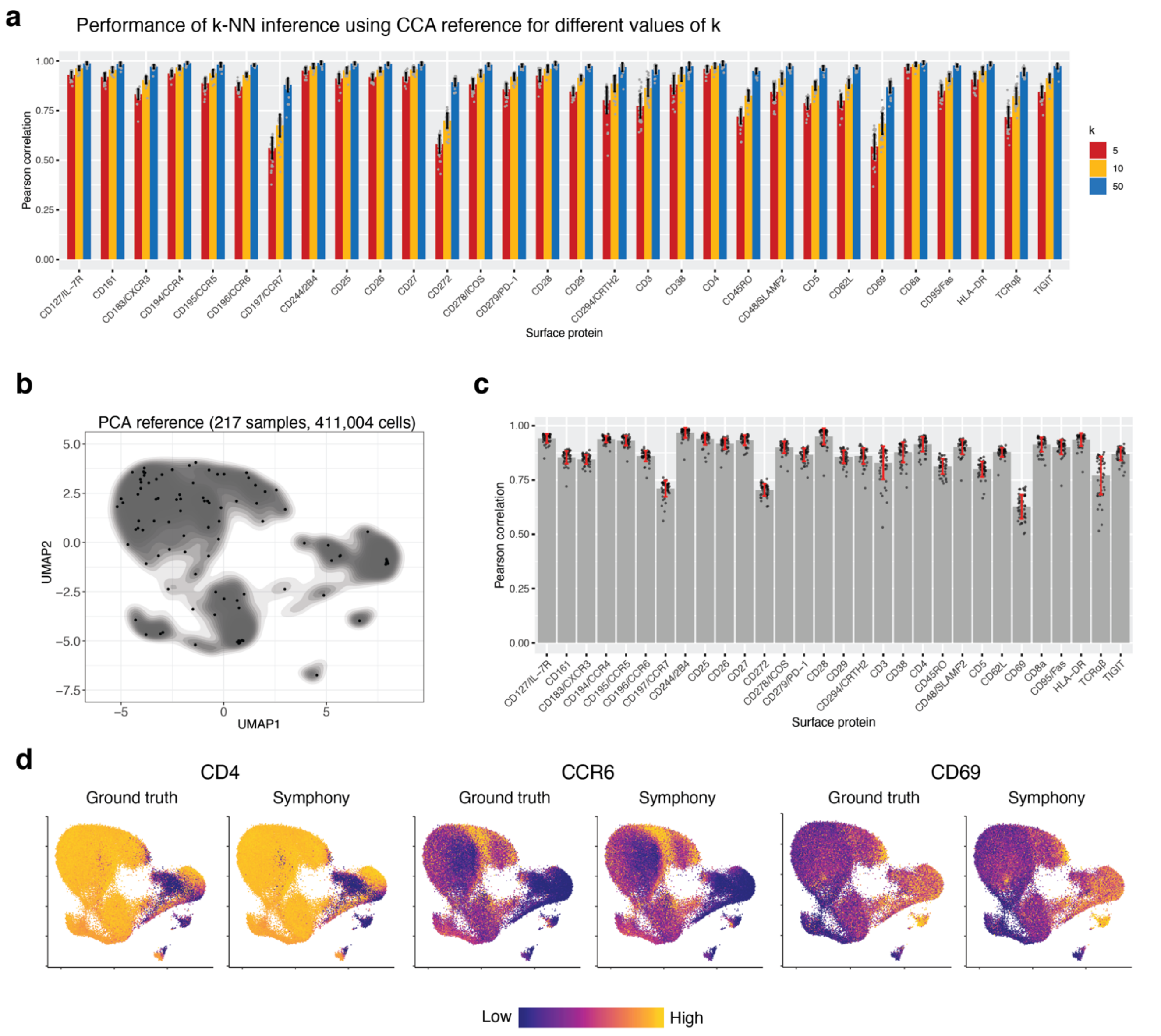

Supplementary Figure 15: Inferring query surface protein expression in memory T cells. (a) Mean Pearson correlation for CCA reference between k-NN predicted protein expression and ground truth for different values of k (total n = 104,716 cells from 54 samples). Bar height represents the mean per-donor correlation for each protein, error bars represent standard deviation, and individual data points (gray) show correlation values per donor. **(b)** Symphony reference built from a standard mRNA PCA embedding (reference protein values were not used to build embedding but treated as annotations only). Contour fill represents density of reference cells. Black points represent soft-cluster centroids in the Symphony mixture model. **(c)** We measured the accuracy of protein expression prediction based on the PCA reference with the Pearson correlation between predicted and ground truth expression for each surface protein across query cells in each donor (total n = 89,085 cells from 54 samples). Note that the number of cells is different from the CCA experiment since a different set of 54 random query samples was selected for each. Bar height represents the mean per-donor correlation for each protein, error bars represent standard deviation, and individual data points show correlation values per donor. **(d)** Ground truth and predicted expression of CD4, CCR6, and CD69 based on PCA reference. Ground truth is the 50-NN-smoothed expression measured in the CITE-seq experiment. Colors are scaled independently for each marker from minimum (blue) to maximum (yellow) expression.

Supplementary Tables 1-11

Supplementary Table 1: Links to public datasets used in the study.

Dataset	URL
10x PBMCs - 5', 3'v1, and 3'v2	Data obtained from Korsunsky et al. (2019):<br>https://github.com/immunogenomics/harmony2019/tree/master/data/figure4<br><br>Original links from 10x:<br>https://support.10xgenomics.com/single-cell-vdj/datasets/2.2.0/vdj_v1_hs_pbmc_5gex<br>https://support.10xgenomics.com/single-cell-gene-expression/datasets/1.1.0/pbmc6k<br>https://support.10xgenomics.com/single-cell-gene-expression/datasets/2.1.0/pbmc8k
PbmcBench	https://zenodo.org/record/3357167#.YSL8p9NKhTY
Pancreas reference - CelSeq, CelSeq2, FluidigmC1, SmartSeq2	Data obtained from Korsunsky et al. (2019):<br>https://github.com/immunogenomics/harmony2019/tree/master/data/figure5<br><br>Links from original studies:<br>https://www.ncbi.nlm.nih.gov/geo/query/acc.cgi?acc=GSE81076<br>https://www.ncbi.nlm.nih.gov/geo/query/acc.cgi?acc=GSE85241<br>https://www.ncbi.nlm.nih.gov/geo/query/acc.cgi?acc=GSE86469<br>https://www.ebi.ac.uk/arrayexpress/experiments/E-MTAB-5061/
Pancreas query - inDrop (Baron et al.)	https://hemberg-lab.github.io/scRNA.seq.datasets/human/pancreas/
Fetal liver – 10x 3prime	https://www.ebi.ac.uk/arrayexpress/experiments/E-MTAB-7407/<br>Note: Data post-doublet removal obtained by contacting Haniffa Lab directly
Fetal liver – 10x 5prime	https://www.ebi.ac.uk/arrayexpress/experiments/E-MTAB-7407/<br>Note: Data post-doublet removal and with updated cell type labels obtained by contacting Haniffa Lab directly
Memory T cell CITE-seq	https://www.ncbi.nlm.nih.gov/geo/query/acc.cgi?acc=GSE158769
Healthy human kidney (fetal)	https://www.kidneycellatlas.org/
Renal cell carcinoma	https://singlecell.broadinstitute.org/single_cell/study/SCP1288/tumor-and-immune-reprogramming-during-immunotherapy-in-advanced-renal-cell-carcinoma#study-summary
Tabula Muris Senis (FACS)	https://figshare.com/articles/dataset/Processed_files_to_use_with_scanpy_/8273102?file=23937842

COVID-19 (1.46 million cells)	Obtained AnnData file GSE158055_covid19.h5ad from: https://drive.google.com/file/d/1TXDJqOvFkJxbcm2u2-_bM5RBdTOqv56w/view, based on Seurat issue: https://github.com/satijalab/seurat/issues/4030<br><br>Original GEO entry: https://www.ncbi.nlm.nih.gov/geo/query/acc.cgi?acc=GSE158055
scATAC-seq hematopoiesis	https://github.com/pinellolab/scATAC-benchmarking/blob/master/Real_Data/Buenrostro_2018/input/combined.sorted.merged.bed

Supplementary Table 2: Canonical lineage markers used to assign cell types for 10x PBMCs. Output from differential expression analysis using 'presto' R package, filtered by AUC > 0.7. feature = gene name; logFC = log fold change between cell in cluster vs. out; statistic = Wilcoxon rank sum U statistic (two-sided); auc = area under ROC curve; pval = nominal p value; padj = Benjamini-Hochberg adjusted p value.

feature	cluster	avgExpr	logFC	statistic	auc	pval	padj
CD3D	0	1.78676830868529	0.877333269978165	37656887.5	0.716988781682442	0	0
CD14	1	1.78477839127833	1.50530784771773	35867022.5	0.850731630640911	0	0
LYZ	1	4.92399328600779	3.5906467827157	39467819.5	0.936139107757723	0	0
IL7R	10	2.04550047000887	1.25311928859376	13579714.5	0.786742365078379	3.13679503276481E-224	3.77468470835634E-221
CD3D	10	1.93635187201459	0.94301151113881	12662328.5	0.733593498706425	2.78494540909109E-135	1.6178612174813E-132
NKG7	10	2.98880961019686	2.41203440799825	15534974	0.900020555379948	0	0
FCGR3A	11	1.94373474844031	1.7555278752743	14362449	0.886365584565502	0	0
GNLY	11	4.22625268218644	3.98632816531166	15847917.5	0.978039933094512	0	0
NKG7	11	4.40615611271639	3.87973883347676	16012681.5	0.988208194731184	0	0
FCER1A	12	2.21268156205806	2.18811766189059	7101564.5	0.953249455123184	0	0
LYZ	12	4.59233650799729	2.92053018185472	6260723.5	0.840382603727941	2.46527467669406E-122	3.75859569939048E-120
CD14	13	1.1540164091107	0.723180724295266	5104550.5	0.728613108054774	2.21657308291791E-86	7.46852134558359E-83
LYZ	13	4.17830950353582	2.49650391370271	5504571.5	0.785711288217196	1.04404287379257E-81	3.19799823541516E-78
MS4A1	15	1.55388485789561	1.19384101525677	2807275	0.846543894142623	6.59199105015374E-119	1.1105527322194E-114
FCER1A	16	1.05950930074463	1.00417568966425	2670357.5	0.852880962380848	0	0
CD4	16	1.19888593683425	0.890821709447361	2557383	0.81679822803367	9.4351522643525E-73	8.36600053671297E-71
PPBP	17	2.9996533286936	2.9802777954512	2981402.5	0.971386415589087	0	0
GNLY	18	4.55629665919862	4.18418355562252	2175569.5	0.960646542379033	4.81086567985013E-156	1.47361189288064E-152
NKG7	18	3.45353005181828	2.79177591228098	2061579.5	0.910313009312962	2.59440869977478E-81	3.23762987889671E-78
CD59	19	0.63224770779975	0.540340970725541	1350437.5	0.746298866048489	7.22323199400776E-66	2.53520394589685E-63
CD3D	19	2.38517541410561	1.35857449873343	1506027.5	0.832283327060927	4.12414220844212E-30	5.24373009702825E-28
IL7R	2	1.93009639836918	1.21411686085724	32187123	0.781712761160087	0	0
CD3D	2	1.94976284046533	1.02574478032896	30977999	0.752347363680327	0	0
CD14	20	1.33284105633847	0.893749918888884	1329227.5	0.769830753094578	8.06174780936685E-31	1.18101100299481E-27
MS4A1	20	1.47104626246811	1.10625303937475	1431703.5	0.829180395088985	7.32216617263723E-57	9.18200198553587E-53

LYZ	20	4.24206810750422	2.52955511750738	1349895	0.781800470159251	6.92642866938767E-21	2.91723859482935E-18
CD59	21	0.765414497853269	0.67231925512772	563984	0.819566692678475	8.17613435994139E-43	1.16731640306723E-40
FCER1A	22	0.782967391361607	0.72144467062165	477699.5	0.738895290833018	2.95557365799568E-48	2.8052140516199E-46
CD34	22	0.725298096778682	0.724647341066981	500362	0.773949157392441	0	0
CD59	22	0.525852657487562	0.432336841491258	497291	0.769199000781123	4.12908544493898E-29	2.73868907444437E-27
CD14	3	1.69313585718211	1.39786966244197	35250510.5	0.856797592679122	0	0
MS4A7	3	0.823124691611784	0.60698401926253	29225816	0.710361593001127	0	0
LYZ	3	4.84438118123862	3.48973699248142	37795908	0.918665929320983	0	0
MS4A1	4	2.26652747634342	2.09228102487396	34444861.5	0.934548042664074	0	0
CD8A	5	1.31956626459957	1.1359604861495	23971531	0.784040114909325	0	0
CD3D	5	2.17559379584735	1.23716036266784	24459894.5	0.800013086124953	0	0
NKG7	5	2.72133993007831	2.21283831969749	25984413.5	0.849875735779722	0	0
CD8A	6	1.31480156739005	1.1153190514941	21368335.5	0.829705518961626	0	0
CD3D	6	1.87974112784284	0.904470643652137	18665587.5	0.724761222670392	4.25357025195161E-186	2.65407029757884E-183
IL7R	7	1.76741135839176	0.974692517036475	15892397	0.729568428269657	8.77053995799087E-182	2.95514573344544E-178
CD3D	7	1.83602038062372	0.848488196606203	15431018	0.708388014020842	7.30751188566763E-136	9.11923353613649E-133
FCGR3A	8	2.37839235614051	2.22538797138707	18217956	0.945813793017875	0	0
MS4A7	8	1.90942728705767	1.70838924767227	17661235	0.916910748095454	0	0
LYZ	8	2.91754494761029	1.25299183653605	13745420	0.7136150634475	1.54685266418214E-124	1.26811809408645E-122
MS4A1	9	2.34422120213461	2.06273196159553	16498005.5	0.929034654591585	0	0

Supplementary Table 3: Top 10 differentially expressed genes (columns) per cluster (rows) used to assign cell types for 10x PBMCs.

Cluster	1st	2nd	3rd	4th	5th	6th	7th	8th	9th	10th
0	RPL32	RPS15A	RPS14	RPS27A	RPS25	RPS3A	RPS12	RPL35A	RPL11	LDHB
1	S100A8	S100A9	S100A6	LYZ	TYROBP	FTL	FCN1	S100A4	GPX1	S100A12
10	CCL5	GZMK	NKG7	GZMA	KLRB1	CTSW	IL32	CST7	KLRG1	LYAR
11	NKG7	PRF1	GNLY	CST7	CTSW	GZMB	GZMA	FGFBP2	KLRD1	CD247
12	HLA-DRB1	HLA-DPB1	HLA-DRA	HLA-DPA1	HLA-DQA1	CST3	FCER1A	HLA-DQB1	CD74	HLA-DMA
13	S100A8	S100A9	LYZ	S100A12	FCN1	FTL	CTSS	S100A6	AIF1	CSTA
14 – dying (removed)	MT-CYB	MT-ATP6	MT-CO2	MALAT1	MT-CO3	MT-ND5	RPL32	MT-ATP8	MT-ND3	MT-ND4L
15	CD79B	CD79A	MS4A1	IGHM	CD37	IGHD	LINC00926	CD74	HLA-DQB1	IGKC
16	ITM2C	LILRA4	IRF7	PLD4	IRF8	JCHAIN	SEC61B	SERPINF1	TCF4	C12orf75
17	PPBP	SDPR	NRGN	HIST1H2AC	PF4	TUBB1	GNG11	GPX1	CLU	SPARC
18	GNLY	CTSW	NKG7	KLRD1	CD7	XCL2	TRDC	HOPX	XCL1	KLRC1
19	ACTG1	PFN1	TMSB10	CORO1A	ACTB	IL32	ARHGDIB	GAPDH	AES	CFL1
2	IL32	LTB	LDHB	IL7R	CD3D	EEF1A1	TRAC	CD3E	CD2	RPSA
20	S100A8	CD79A	MS4A1	S100A9	CD74	CD79B	IGHM	HLA-DRB1	MNDA	LINC00926
21	JCHAIN	MZB1	PPIB	SEC11C	ITM2C	TNFRSF17	HSP90B1	SSR4	SUB1	IGHA1
22	PRSS57	SERPINB1	HNRNPA1	RP11-620J15.3	NPM1	SOX4	CAT	RPS24	GNB2L1	STMN1
3	LYZ	CTSS	FCN1	CST3	TYROBP	GPX1	PSAP	FTH1	S100A9	GSTP1
4	CD79A	CD74	CD79B	CD37	TCL1A	MS4A1	IGHM	HLA-DRA	HLA-DPB1	HLA-DQB1
5	CCL5	NKG7	IL32	CTSW	B2M	HLA-A	CST7	HLA-C	CD3D	GZMA
6	CD8B	CD8A	RPL32	RPS14	RPS12	RPS3A	RP11-291B21.2	RPS15A	RPS25	RPS5
7	RPS15A	RPS27A	RPS12	RPL32	LDHB	RPS14	RPL11	RPS25	RPS3	EEF1A1
8	LST1	COTL1	AIF1	FCER1G	FCGR3A	FTL	SAT1	PSAP	FTH1	LINC01272
9	MS4A1	CD79A	CD74	CD79B	BANK1	CD37	HLA-DQA1	HLA-DRA	HLA-DPB1	HLA-DPA1

Supplementary Table 4: Cell type classification confusion matrix for the three 10x PBMCs mapping experiments.

True cell type	Query dataset	Predicted B	Predicted DC	Predicted HSC	Predicted MK	Predicted Mono_CD14	Predicted Mono_CD16	Predicted NK	Predicted T_CD4	Predicted T_CD8
B	3'v1	587	0	0	0	0	0	1	0	1
DC	3'v1	0	77	0	0	0	1	0	0	0
HSC	3'v1	4	0	17	0	0	0	0	0	0
MK	3'v1	0	0	0	26	2	2	0	0	0
Mono_CD14	3'v1	0	5	0	0	800	36	0	0	0
Mono_CD16	3'v1	0	0	0	0	3	349	0	0	0
NK	3'v1	0	0	0	0	0	0	285	0	6
T_CD4	3'v1	0	0	0	0	0	0	0	1639	18
T_CD8	3'v1	0	0	0	0	0	0	7	88	804
B	3'v2	1226	0	0	1	0	0	2	0	2
DC	3'v2	0	247	0	0	8	0	0	0	0
HSC	3'v2	0	0	18	0	1	0	0	0	0
MK	3'v2	0	0	0	59	2	1	0	1	0
Mono_CD14	3'v2	0	2	0	0	1820	0	0	0	0
Mono_CD16	3'v2	0	0	0	0	16	226	0	0	0
NK	3'v2	0	0	0	0	0	0	321	0	1
T_CD4	3'v2	0	0	0	0	0	0	0	2480	44
T_CD8	3'v2	0	0	0	0	0	0	29	110	1688
B	5'	1174	0	0	0	0	0	0	0	0
DC	5'	0	177	0	0	4	0	0	0	0
HSC	5'	2	0	22	0	0	0	0	0	0
MK	5'	0	0	0	54	1	0	0	0	0
Mono_CD14	5'	1	1	0	0	2220	2	0	1	2
Mono_CD16	5'	0	1	0	0	26	346	0	0	0
NK	5'	1	0	0	0	0	0	299	1	2
T_CD4	5'	0	0	0	0	0	0	0	2092	43
T_CD8	5'	0	0	0	0	0	0	0	41	995

Supplementary Table 5: Runtime benchmark comparing Symphony, scArches, and Seurat reference mapping. The pipelines were run on different-sized reference and query datasets (num cells). Elapsed time for reference building (RB), query mapping (QM), or de novo integration (DN) was measured in seconds (s). Grey shading indicates job failed due to excess memory or time requirements. MEM = memory error (>120 GB), TIME = elapsed time exceeded >24 hrs. Note: All jobs run on a Linux cluster (CPUs). Symphony/Harmony and Seurat were run using 4 cores; scArches/trVAE was run with 48 cores.

Query num cells	Ref num cells	Symphony RB (s)	Symphony QM (s)	Harmony DN (s)	scArches RB (s)	scArches QM (s)	trVAE DN (s)	Seurat RB (s)	Seurat QM (s)	Seurat DN (s)
1000	20000	61.667	0.056	167.558	919.205	4.980392	698.4887	754.823	19.963	894.035
1000	50000	419.6	0.163	490.889	8908.704	8.132626	6240.8065	5484.759	49.384	6926.44
1000	100000	1411.927	0.082	391.786	61663.67	21.394529	50170.357	MEM	MEM	MEM
1000	250000	4374.974	0.07	7058.002	TIME	TIME	TIME	MEM	MEM	MEM
1000	500000	21093.27	0.195	8895.052	TIME	TIME	TIME	MEM	MEM	MEM
10000	20000	177.539	2.249	67.431	596.6031	100.05232	1866.4176	768.275	133.855	1874.4
10000	50000	447.894	1.996	493.657	5629.113	142.92039	12379.71	6080.779	321.077	MEM
10000	100000	990.855	1.382	1459.878	40529.68	156.72127	80063.65	MEM	MEM	MEM
10000	250000	6775.721	1.715	4117.026	TIME	TIME	TIME	MEM	MEM	MEM
10000	500000	10189.41	1.18	10243.75	TIME	TIME	TIME	MEM	MEM	MEM
100000	20000	173.493	43.261	512.148	727.9022	38120.236	67454.434	784.617	1486.463	MEM
100000	50000	163.667	13.535	2323.836	12598.06	70715.102	TIME	5704.96	3323.411	MEM
100000	100000	485.216	13.401	1074.483	TIME	TIME	TIME	MEM	MEM	MEM
100000	250000	3972.34	31.683	10031.27	TIME	TIME	TIME	MEM	MEM	MEM
100000	500000	16781.98	46.042	16520.88	TIME	TIME	TIME	MEM	MEM	MEM

Supplementary Table 6: Effect of number of query cells and query donors on mapping runtime. Elapsed time is shown (in s). Boldface visually highlights the parameters being tested (varied) for a given series of experiments. k = 100 centroids, d = 20 dimensions, mapping to a 50,000-cell reference (built from 30 donors) for all experiments.

Query num cells	Query num donors	Query mapping elapsed time (s)
Effect of # query donors (keep num cells constant)		
10000	**6**	0.81
10000	**15**	1.861
10000	**30**	3.887
10000	**60**	1.196
10000	**120**	1.536
Effect of # query cells (keep num donors constant)		
**1000**	6	0.067
**2500**	6	0.189
**5000**	6	0.393
**10000**	6	0.81

Supplementary Table 7: Effect of number of reference centroids and embedding dimensions on runtime. Elapsed time is shown (in s). Boldface visually highlights the parameters being tested (varied) for a given series of experiments. 50,000-cell reference (30 donors) and 10,000-cell query (6 donors) used for all experiments.

k (# centroids)	d (# dimensions)	Reference building elapsed time (s)	Query mapping elapsed time (s)
Effect of # centroids (keep everything else constant)			
**25**	20	58.94	0.693
**50**	20	70.89	0.741
**100**	20	139.38	0.805
**200**	20	275.89	0.983
**400**	20	1781.44	5.106
Effect of # dimensions (keep everything else constant)			
100	**10**	219.04	0.753
100	**20**	142.43	0.812
100	**40**	270.97	1.581
100	**80**	176.37	0.934
100	**160**	300.96	1.132
100	**320**	567.17	1.36

Supplementary Table 8: Cell type classification confusion matrix for human cells in pancreas benchmarking example. True cell types were defined by the original authors (Baron et al., 2016). Predicted labels were assigned using different reference mapping methods (and alternative annotation transfer methods for Seurat).

True cell type	Method	Predicted acinar	Predicted alpha	Predicted beta	Predicted delta	Predicted ductal	Predicted endothelial	Predicted epsilon	Predicted gamma	Predicted immune	Predicted stellate
acinar	Symphony (5-NN)	950	0	0	0	2	1	0	0	0	5
alpha	Symphony (5-NN)	18	2295	5	1	1	0	0	2	2	2
beta	Symphony (5-NN)	21	8	2481	9	2	0	0	3	0	1
delta	Symphony (5-NN)	4	1	33	559	1	0	0	0	2	1
ductal	Symphony (5-NN)	176	1	4	0	896	0	0	0	0	0
endothelial	Symphony (5-NN)	0	0	0	0	0	247	0	0	0	5
epsilon	Symphony (5-NN)	0	2	0	6	0	0	8	2	0	0
gamma	Symphony (5-NN)	1	4	4	0	0	0	0	246	0	0
immune	Symphony (5-NN)	0	1	0	0	4	0	0	0	82	0
stellate	Symphony (5-NN)	0	1	1	0	0	0	0	0	0	455
acinar	Seurat (5-NN)	951	0	0	0	1	1	0	0	0	5
alpha	Seurat (5-NN)	12	2303	4	1	3	0	0	2	1	0
beta	Seurat (5-NN)	16	19	2483	5	0	0	0	2	0	0
delta	Seurat (5-NN)	2	4	64	529	0	0	0	0	2	0
ductal	Seurat (5-NN)	178	1	5	0	893	0	0	0	0	0
endothelial	Seurat (5-NN)	0	0	6	0	7	235	0	0	0	4
epsilon	Seurat (5-NN)	0	4	1	5	0	0	4	4	0	0
gamma	Seurat (5-NN)	1	6	3	0	1	0	0	244	0	0
immune	Seurat (5-NN)	0	0	0	0	7	0	0	0	80	0
stellate	Seurat (5-NN)	0	4	3	1	3	0	0	0	0	446
acinar	Seurat (TransferData)	951	0	0	0	2	0	0	0	0	5
alpha	Seurat (TransferData)	8	2310	4	1	0	0	0	2	1	0
beta	Seurat (TransferData)	18	10	2489	6	0	0	0	2	0	0
delta	Seurat (TransferData)	2	3	56	538	0	0	0	0	2	0
ductal	Seurat (TransferData)	177	1	6	1	892	0	0	0	0	0
endothelial	Seurat (TransferData)	0	0	7	0	6	235	0	0	0	4

epsilon	Seurat (TransferData)	0	0	1	9	0	0	0	8	0	0
gamma	Seurat (TransferData)	1	1	3	0	0	0	0	250	0	0
immune	Seurat (TransferData)	0	1	3	0	2	0	0	0	81	0
stellate	Seurat (TransferData)	0	4	5	1	1	0	0	0	0	446
acinar	scArches/trVAE (5-NN)	144	293	465	2	48	0	0	0	0	6
alpha	scArches/trVAE (5-NN)	2	1724	201	370	2	0	4	23	0	0
beta	scArches/trVAE (5-NN)	2	174	2186	159	2	0	0	2	0	0
delta	scArches/trVAE (5-NN)	0	19	14	565	2	0	0	1	0	0
ductal	scArches/trVAE (5-NN)	171	26	30	12	830	0	2	3	0	3
endothelial	scArches/trVAE (5-NN)	0	18	8	26	38	58	0	1	0	103
epsilon	scArches/trVAE (5-NN)	0	9	0	9	0	0	0	0	0	0
gamma	scArches/trVAE (5-NN)	0	18	33	63	1	0	2	138	0	0
immune	scArches/trVAE (5-NN)	0	28	9	19	6	0	1	2	22	0
stellate	scArches/trVAE (5-NN)	0	11	31	7	9	0	0	0	0	399

Supplementary Table 9: Cell type classification confusion matrix for mouse cells in pancreas benchmarking example. True cell types were defined by the original authors (Baron et al., 2016). Predicted labels were assigned using different reference mapping methods (and alternative annotation transfer methods for Seurat).

True cell type	Method	Predicted acinar	Predicted alpha	Predicted beta	Predicted delta	Predicted ductal	Predicted endothelial	Predicted epsilon	Predicted gamma	Predicted immune	Predicted stellate
alpha	Symphony (5-NN)	0	123	56	9	0	0	0	3	0	0
beta	Symphony (5-NN)	0	7	861	25	0	0	0	1	0	0
delta	Symphony (5-NN)	0	3	29	185	0	0	0	1	0	0
ductal	Symphony (5-NN)	2	0	2	0	270	0	0	0	0	1
endothelial	Symphony (5-NN)	0	0	0	0	0	139	0	0	0	0
gamma	Symphony (5-NN)	0	4	18	5	0	0	0	14	0	0
immune	Symphony (5-NN)	1	0	1	0	1	0	0	0	58	0
stellate	Symphony (5-NN)	0	0	0	0	0	1	0	0	1	59
alpha	Seurat (5-NN)	0	161	17	6	0	0	3	4	0	0
beta	Seurat (5-NN)	0	28	850	15	0	0	1	0	0	0
delta	Seurat (5-NN)	0	12	38	156	0	0	6	6	0	0
ductal	Seurat (5-NN)	2	1	2	0	270	0	0	0	0	0
endothelial	Seurat (5-NN)	0	5	2	0	2	129	0	0	0	1
gamma	Seurat (5-NN)	0	5	11	2	0	0	3	20	0	0
immune	Seurat (5-NN)	1	0	1	0	5	0	0	0	54	0
stellate	Seurat (5-NN)	0	0	0	0	0	1	0	0	1	59
alpha	Seurat (TransferData)	0	150	31	8	0	0	0	2	0	0
beta	Seurat (TransferData)	0	18	856	20	0	0	0	0	0	0
delta	Seurat (TransferData)	0	6	36	167	0	0	1	8	0	0
ductal	Seurat (TransferData)	1	0	2	0	272	0	0	0	0	0
endothelial	Seurat (TransferData)	0	4	2	0	1	129	0	0	0	3
gamma	Seurat (TransferData)	0	6	13	6	0	0	0	16	0	0
immune	Seurat (TransferData)	1	0	2	0	0	0	0	0	58	0
stellate	Seurat (TransferData)	0	0	0	0	0	1	0	0	0	60
alpha	scArches/trVAE (5-NN)	0	191	0	0	0	0	0	0	0	0

beta	scArches/trVAE (5-NN)	0	826	68	0	0	0	0	0	0	0
delta	scArches/trVAE (5-NN)	0	205	7	6	0	0	0	0	0	0
ductal	scArches/trVAE (5-NN)	35	171	10	1	58	0	0	0	0	0
endothelial	scArches/trVAE (5-NN)	0	78	0	0	0	60	0	0	0	1
gamma	scArches/trVAE (5-NN)	0	38	2	1	0	0	0	0	0	0
immune	scArches/trVAE (5-NN)	1	58	0	0	0	0	0	0	0	2
stellate	scArches/trVAE (5-NN)	0	47	0	0	0	0	0	0	0	14

Supplementary Table 10: LISI comparison between methods. Degree of mixing as measured by Local Inverse Simpson's Index (LISI) calculated between reference and query cells and between donors within the query for reference mapping and corresponding *de novo* integration methods for pancreas benchmarking example. ref_query LISI: degree of mixing between reference and query cells (ranges from 1-2); query_donors LISI: degree of mixing among query donors (ranges from 1-6).

Baron et al. human cells		
Method	mean ref_query LISI	mean query_donors LISI
Symphony mapping	1.51	2.67
Harmony de novo	1.4	2.55
Seurat mapping	1.17	2.04
Seurat de novo	1.38	2.96
scArches mapping	1.02	1.12
trVAE de novo	1.27	2.52
Baron et al. mouse cells		
Method	mean ref_query LISI	mean query_donors LISI
Symphony mapping	1.26	2.91
Harmony de novo	1.23	2.7
Seurat mapping	1.05	2.46
Seurat de novo	1.2	3.09
scArches mapping	1	1.24
trVAE de novo	1.19	3.05

Supplementary Table 11: Cell type classification confusion matrix for fetal liver example. Mapping query (10x 5') cells onto the reference (10x 3') atlas of fetal liver cells, prediction using 10-NN. True cell types as defined by Popescu et al. (2019) (rows) and Symphony predicted annotations (columns).

	B cell	DC precursor	DC1	DC2	Early Erythroid	Early lymphoid/T	Endothelial cell	Fibroblast	Hepatocyte	HSC_MPP	ILC precursor	Kupffer Cell	Late Erythroid	Mast cell	Megakaryocyte	MEMP	Mid Erythroid	Mono-Mac	Monocyte	Monocyte precursor	Neut-myeloid prog.	NK	pDC precursor	Pre pro B cell	pre-B cell	pro-B cell	VCAM1+ EI macro.
B cell	494	0	0	0	1	4	0	0	0	3	0	0	0	0	0	0	0	1	0	0	0	5	3	0	76	0	0
DC precursor	0	91	8	57	0	0	0	0	0	0	0	1	0	0	0	0	0	0	56	73	21	0	2	0	0	0	0
DC1	0	0	73	2	0	0	0	0	0	0	0	0	0	0	0	0	0	4	0	0	0	0	0	0	0	0	0
DC2	0	2	18	1251	0	0	0	0	0	0	0	0	0	0	0	0	0	280	45	0	0	0	7	0	0	0	0
Early Erythroid	0	0	0	0	1417	0	0	0	0	0	0	0	0	4	0	46	129	0	0	0	0	0	0	0	0	0	0
Early lymphoid/T	1	0	0	0	0	304	0	0	0	0	350	0	0	0	0	0	0	0	0	0	0	30	0	0	0	0	0
Endothelial cell	1	0	0	0	0	0	563	0	0	1	0	0	0	0	0	0	1	0	0	0	0	0	0	0	1	0	0
Fibroblast	0	0	0	0	0	0	6	53	0	0	0	0	1	0	0	0	0	0	0	0	0	0	0	0	0	0	0
Hepatocyte	0	0	0	0	0	0	0	0	87	0	0	0	0	0	0	0	0	0	0	0	0	0	0	0	0	0	0
HSC_MPP	0	0	0	0	0	2	0	1	0	531	0	0	0	0	0	3	0	0	0	0	52	0	0	11	0	5	0
ILC precursor	0	0	0	0	0	0	0	0	0	0	153	0	0	0	0	0	0	0	0	0	0	1	0	0	0	0	0
Kupffer Cell	2	0	0	0	0	0	8	0	0	0	0	5064	0	0	0	0	0	111	2	0	0	0	4	1	0	0	0
Late Erythroid	0	0	0	0	0	0	0	0	0	0	0	0	141	0	0	0	2	0	0	0	0	1	0	0	0	0	0
Mast cell	1	0	0	0	9	0	0	0	0	2	0	0	0	532	0	0	0	0	0	0	0	0	0	0	0	0	0
Megakaryocyte	0	0	0	0	0	0	0	0	0	0	0	0	0	0	357	0	0	0	0	0	0	0	0	0	0	1	0
MEMP	0	0	0	0	12	0	0	0	0	29	0	0	0	3	98	165	0	0	0	0	0	0	0	0	0	0	0
Mid Erythroid	0	0	0	0	104	0	0	0	0	0	0	1	32	0	0	0	2043	0	0	0	0	2	0	0	0	1	9
Mono-Mac	0	0	1	13	0	0	0	0	0	0	0	70	0	0	0	0	0	992	6	0	2	0	2	0	0	0	0
Monocyte	1	0	0	98	0	0	0	0	0	0	0	0	0	1	0	0	0	40	907	10	1	0	0	0	0	0	0
Monocyte precursor	0	50	3	28	0	0	0	0	0	0	0	0	0	0	0	0	0	4	12	37	4	0	0	0	0	0	0
Neut-myeloid prog.	0	0	0	0	0	0	0	0	0	0	1	0	0	0	0	0	0	0	0	0	54	0	0	0	0	0	0
NK	0	0	0	0	0	10	0	0	0	0	1	0	0	0	0	0	0	0	1	0	0	1780	0	0	0	0	0
pDC precursor	0	0	1	0	0	0	0	0	0	0	0	1	0	0	0	0	0	0	0	0	1	1	66	0	0	0	0
Pre pro B cell	0	0	0	0	0	0	0	0	0	0	0	0	0	0	0	0	0	0	0	0	0	0	1	65	10	10	0
pre-B cell	71	0	0	0	0	8	0	0	0	0	0	0	0	0	0	0	1	0	0	0	3	4	4	3	702	463	0
pro-B cell	0	0	0	0	0	0	0	0	0	0	0	0	0	0	0	0	0	0	0	0	0	0	0	12	25	272	0
VCAM1+ EI macro.	0	0	0	0	0	0	0	0	0	0	0	442	0	0	0	0	0	42	0	0	0	0	0	0	0	0	8

Supplementary Methods

During Symphony reference building, we calculate two reference compression terms, $\mathbf{N_r}$ and $\mathbf{C}$, which are precomputed in advance to be used later during the correction step of the reference mapping algorithm. This section describes the relevant parts of the linear mixture model framework shared by Harmony and Symphony and derives the reference compression terms.

Harmony mixture of experts model learned from reference cells

In the Harmony mixture model learned during reference integration, the k clusters represent k "experts" in a mixture of experts that serve as surrogate variables for cell states within the low-dimensional space. For each reference cluster k, we learn a cluster-specific linear model for each PC: that is, the location in PC space for each reference cell i ($\mathbf{Z_{r[\cdot,i]}}$) can be modeled as in Equation (1). For each cluster k, we estimated $\mathbf{B_{rk}} \in \mathbb{R}^{(1+b)\times d}$, representing the parameters of the linear model. The batch-independent intercept terms $\mathbf{B_{rk[0,\cdot]}}$ represent the location of cluster centroid k in PC space. The remaining batch-dependent terms $\mathbf{B_{rk[1:b,\cdot]}}$ represent reference batch effect coefficients for each PC. See Korsunsky et al. (2019)[6] for full details.

$$\mathbf{Z_{r[\cdot,i]}} = \sum_k \mathbf{R_{r[k,i]}} \left[\mathbf{B^T_{rk[0,\cdot]}} + \mathbf{B^T_{rk[1:b,\cdot]}}\mathbf{X_r}\right] + \varepsilon \tag{1}$$

After Harmony integration, the batch effects for the reference cells have been removed by subtracting the batch-dependent terms from each cell. In the final integrated embedding, the harmonized PCs for each reference cell are thereby modeled as the weighted summation of only the intercept terms for the clusters over which the cell is assigned (captured in $\mathbf{R_r}$) as well as a cell-specific residual ε.

$$\hat{\mathbf{Z}}_{\mathbf{r[\cdot,i]}} = \sum_k \mathbf{R_{r[k,i]}}\, \mathbf{B^T_{rk[0,\cdot]}} + \varepsilon \tag{2}$$

Symphony models non-harmonized query cells with harmonized reference cells

The goal of reference mapping is to add the query cells to our model, modeling all cells together in order to estimate and remove the query batch effects. Let $N = m + n$ represent the total number of cells (sum of number of query and reference cells). Let $\mathbf{X}^* \in [0,1]^{(1+c)\times N}$ denote a design matrix for reference mapping in which the first m columns represent query cells, and the remaining n columns represent harmonized reference cells. The star ($*$) indicates the design matrix has been augmented: the first row ($\mathbf{X_{[0,\cdot]}}$) consists entirely of 1s, corresponding to the batch-independent intercepts (we model the intercepts for all cells). The remaining c rows ($\mathbf{X^*_{[1:c,\cdot]}}$) represent the one-hot batch assignment of the cells among the c query batches. Note that for the

reference cell columns, these values are all 0 since the reference cells do not belong to any *query* batches. We do not include reference batch terms in our design matrix because the reference batch-dependent factors have already been removed during reference integration. Therefore, each harmonized reference cell is modeled only by a weighted average of the centroid locations for the clusters over which it belongs and a cell-specific residual.

Let matrix $\mathbf{R} \in \mathbb{R}^{k\times N}$ denote the assignment of query and reference cells (columns) across the reference clusters (rows). Then, the parameters ($\mathbf{B_{qk}}$) of the mixture of experts model can be solved for as in Equation (3). The notation $\mathrm{diag}(\mathbf{R_k}) \in \mathbb{R}^{N\times N}$ denotes the diagonalized kth row of $\mathbf{R}$. Let $\mathbf{Z} \in \mathbb{R}^{d\times N}$ denote the horizontal matrix concatenation of the uncorrected query cells in original PC space ($\mathbf{Z_q}$) and corrected reference cells in harmonized space ($\hat{\mathbf{Z}}_\mathbf{r}$). For each cluster k, let matrix $\mathbf{B_{qk}} \in \mathbb{R}^{(1+c)\times d}$ represent the query parameters to be estimated. The first row of $\mathbf{B_{qk}}$ represents the batch-independent intercept terms, and the remaining c rows of $\mathbf{B_{qk}}$ represent the query batch-dependent coefficients to be estimated.

$$\mathbf{B_{qk}} \approx \left(\mathbf{X}^* \,\mathrm{diag}(\mathbf{R_k})\, \mathbf{X}^{*\mathbf{T}} + \lambda \mathbf{I}\right)^{-1} \mathbf{X}^* \,\mathrm{diag}(\mathbf{R_k})\, \mathbf{Z}^\mathbf{T} \tag{3}$$

Derivation of cached reference-dependent terms

Instead of directly solving Eq. (3) above, we rewrite $\mathrm{diag}(\mathbf{R_k})$, $\mathbf{X}^*$, and $\mathbf{Z}$ by separating out the reference and query-dependent components of each matrix. This allows us to determine which components of the calculation can be precomputed during reference building to reduce computational steps during reference mapping. Assuming the query cells are placed in the first m columns of $\mathbf{R}$ and the reference cells are placed in the last n columns of $\mathbf{R}$, then $\mathbf{R}$ is the horizontal concatenation of $\mathbf{R_q}$ and $\mathbf{R_r}$. Let vector $\mathbf{R_{q[k,\cdot]}}$ of size m denote the kth row of $\mathbf{R_q}$, and let $\mathbf{R_q^{(k)}}$ denote the diagonalized square matrix (of dimensions $m \times m$) of $\mathbf{R_{q[k,\cdot]}}$. Let vector $\mathbf{R_{r[k,\cdot]}}$ of size n denote the kth row of $\mathbf{R_r}$, and let $\mathbf{R_r^{(k)}}$ denote the diagonalized square matrix (of dimensions $n \times n$) of $\mathbf{R_{r[k,\cdot]}}$. Then, $\mathrm{diag}(\mathbf{R_k})$ can be rewritten as $\mathbf{R_q^{(k)}} \oplus \mathbf{R_r^{(k)}}$, the direct sum of the diagonal matrices for the query and reference cells.

$$\mathrm{diag}(\mathbf{R_k}) = \mathbf{R_q^{(k)}} \oplus \mathbf{R_r^{(k)}} = \begin{bmatrix} \mathbf{R_{[k,1]}} & \cdots & 0 \\ \vdots & \ddots & \vdots \\ 0 & \cdots & \mathbf{R_{[k,m+n]}} \end{bmatrix}$$

$$\mathbf{R_q^{(k)}} = \begin{bmatrix} \mathbf{R_{q[k,1]}} & 0 & 0 \\ 0 & \ddots & 0 \\ 0 & 0 & \mathbf{R_{q[k,m]}} \end{bmatrix} \qquad \mathbf{R_r^{(k)}} = \begin{bmatrix} \mathbf{R_{r[k,1]}} & 0 & 0 \\ 0 & \ddots & 0 \\ 0 & 0 & \mathbf{R_{q[k,n]}} \end{bmatrix}$$

Similarly, we can partition the full design matrix $\mathbf{X}^*$ into the left m columns and right n columns that represent the query and reference components: $\mathbf{X}_\mathbf{q}^* \in \{0,1\}^{(1+c)\times m}$ and $\mathbf{X}_\mathbf{r}' \in \{0,1\}^{(1+c)\times n}$. The horizontal concatenation of $\mathbf{X}_\mathbf{q}^*$ and $\mathbf{X}_\mathbf{r}'^*$ yields $\mathbf{X}^*$. Note that $\mathbf{X}_\mathbf{r}'$ is not the original reference design matrix across reference batches ($\mathbf{X}_\mathbf{r}$), but rather assignment of reference cells (columns) to *query* batches (rows). Since reference cells do not belong to any query batches, $\mathbf{X}_\mathbf{r}'$ is a zero matrix, and $\mathbf{X}_\mathbf{r}'^*$ is the same zero matrix augmented with a row of 1s. In a simple example where there are two batches in the query (c = 2), the design matrices take the form:

$$\mathbf{X}^* = \begin{bmatrix} 1 \cdots 1 & 1 \\ \mathbf{X_q} & \mathbf{X}_\mathbf{r}' \end{bmatrix} = \begin{bmatrix} 1 & \cdots & 1\,1 & \cdots & 1 \\ 1 & \cdots & 0\,0 & \cdots & 0 \\ 0 & \cdots & 1\,0 & \cdots & 0 \end{bmatrix}$$

$$\mathbf{X}_\mathbf{q}^* = \begin{bmatrix} 1 & \cdots & 1 \\ 1 & \cdots & 0 \\ 0 & \cdots & 1 \end{bmatrix} \qquad \mathbf{X}_\mathbf{r}'^* = \begin{bmatrix} 1 & \cdots & 1 \\ 0 & \cdots & 0 \\ 0 & \cdots & 0 \end{bmatrix}$$

Similarly, we can partition the embedding $\mathbf{Z}$ into the left m columns and right n columns that represent the query and reference components: $\mathbf{Z_q} \in \mathbb{R}^{d\times m}$ and $\hat{\mathbf{Z}}_\mathbf{r} \in \mathbb{R}^{d\times n}$, respectively. The horizontal concatenation of $\mathbf{Z_q}$ and $\hat{\mathbf{Z}}_\mathbf{r}$ yields $\mathbf{Z}$.

$$\mathbf{Z_q} = \begin{bmatrix} \mathbf{Z_{q[1,1]}} & \cdots & \mathbf{Z_{q[1,m]}} \\ \vdots & \ddots & \vdots \\ \mathbf{Z_{q[d,1]}} & \cdots & \mathbf{Z_{q[d,m]}} \end{bmatrix} \qquad \hat{\mathbf{Z}}_\mathbf{r} = \begin{bmatrix} \hat{\mathbf{Z}}_\mathbf{r[1,1]} & \cdots & \hat{\mathbf{Z}}_\mathbf{r[1,n]} \\ \vdots & \ddots & \vdots \\ \hat{\mathbf{Z}}_\mathbf{r[d,1]} & \cdots & \hat{\mathbf{Z}}_\mathbf{r[d,n]} \end{bmatrix}$$

We can then explicitly substitute the query-dependent and reference-dependent components of $\mathrm{diag}(\mathbf{R_k})$, $\mathbf{X}^*$, and $\mathbf{Z}$ to rewrite Equation (3) as follows.

$$\mathbf{B_{qk}} = \left([\mathbf{X}_\mathbf{q}^* \quad \mathbf{X}_\mathbf{r}'^*] \begin{bmatrix} \mathbf{R}_\mathbf{q}^{(\mathbf{k})} & 0 \\ 0 & \mathbf{R}_\mathbf{r}^{(\mathbf{k})} \end{bmatrix} \begin{bmatrix} \mathbf{X}_\mathbf{q}^\mathbf{T} \\ \mathbf{X}_\mathbf{r}^\mathbf{T} \end{bmatrix} + \lambda\mathbf{I} \right)^{-1} [\mathbf{X}_\mathbf{q}^* \quad \mathbf{X}_\mathbf{r}'^*] \begin{bmatrix} \mathbf{R}_\mathbf{q}^{(\mathbf{k})} & 0 \\ 0 & \mathbf{R}_\mathbf{r}^{(\mathbf{k})} \end{bmatrix} \begin{bmatrix} \mathbf{Z}_\mathbf{q}^\mathbf{T} \\ \hat{\mathbf{Z}}_\mathbf{r}^\mathbf{T} \end{bmatrix}$$

$$\mathbf{B_{qk}} = \left([\mathbf{X}_\mathbf{q}^*\mathbf{R}_\mathbf{q}^{(\mathbf{k})} \quad \mathbf{X}_\mathbf{r}'^*\mathbf{R}_\mathbf{r}^{(\mathbf{k})}] \begin{bmatrix} \mathbf{X}_\mathbf{q}^\mathbf{T} \\ \mathbf{X}_\mathbf{r}^\mathbf{T} \end{bmatrix} + \lambda\mathbf{I} \right)^{-1} [\mathbf{X}_\mathbf{q}^*\mathbf{R}_\mathbf{q}^{(\mathbf{k})} \quad \mathbf{X}_\mathbf{r}'^*\mathbf{R}_\mathbf{r}^{(\mathbf{k})}] \begin{bmatrix} \mathbf{Z}_\mathbf{q}^\mathbf{T} \\ \hat{\mathbf{Z}}_\mathbf{r}^\mathbf{T} \end{bmatrix}$$

$$\mathbf{B_{qk}} = \left(\left[\mathbf{X}_\mathbf{q}^*\mathbf{R}_\mathbf{q}^{(\mathbf{k})}\mathbf{X}_\mathbf{q}^{*\mathbf{T}} + \mathbf{X}_\mathbf{r}'^*\mathbf{R}_\mathbf{r}^{(\mathbf{k})}\mathbf{X}_\mathbf{r}'^{*\mathbf{T}} \right] + \lambda\mathbf{I} \right)^{-1} \left[\mathbf{X}_\mathbf{q}^*\mathbf{R}_\mathbf{q}^{(\mathbf{k})}\mathbf{Z}_\mathbf{q}^\mathbf{T} + \mathbf{X}_\mathbf{r}'^*\mathbf{R}_\mathbf{r}^{(\mathbf{k})}\hat{\mathbf{Z}}_\mathbf{r}^\mathbf{T} \right] \quad (4)$$

In Equation (4), the bolded terms designate terms that depend only on reference cells and can therefore be precomputed ahead of time during the reference building process and subsequently cached for later use during query mapping. The first of these terms, $\mathbf{X}_\mathbf{r}'^*\mathbf{R}_\mathbf{r}^{(\mathbf{k})}\mathbf{X}_\mathbf{r}'^{*\mathbf{T}}$ of dimensions $(1+c)\times(1+c)$, can be further simplified as follows.

$$\mathbf{X_r'^{*}R_r^{(k)}X_r'^{*T}} = \begin{bmatrix} 1 & \cdots & 1 \\ 0 & \cdots & 0 \\ 0 & \cdots & 0 \end{bmatrix} \mathbf{R_r^{(k)}} \begin{bmatrix} 1 & 0 & 0 \\ \vdots & \vdots & \vdots \\ 1 & 0 & 0 \end{bmatrix}$$

$$= \begin{bmatrix} \mathbf{R_{r[k,1]}} & \cdots & \mathbf{R_{r[k,n]}} \\ 0 & \cdots & 0 \\ 0 & \cdots & 0 \end{bmatrix} \begin{bmatrix} 1 & 0 & 0 \\ \vdots & \vdots & \vdots \\ 1 & 0 & 0 \end{bmatrix} = \begin{bmatrix} \sum_{\mathbf{i=1}}^{\mathbf{n}} \mathbf{R_{r[k,i]}} & 0 & 0 \\ 0 & 0 & 0 \\ 0 & 0 & 0 \end{bmatrix} = \begin{bmatrix} \mathbf{N_k} & 0 & 0 \\ 0 & 0 & 0 \\ 0 & 0 & 0 \end{bmatrix}$$

Intuitively, $\mathbf{N_k} \in \mathbb{R}$ is the number of cells (can be a non-integer number since the cells are soft assigned) belonging to cluster k. Therefore, to capture this term for all k clusters, we need only save $\mathbf{N_r} \in \mathbb{R}^{k\times 1}$, a vector containing the size of each of the k clusters in terms of the number of cells contained within them. Similarly, the second of the reference-dependent terms, $\mathbf{X_r'^{*}R_r^{(k)}\hat{Z}_r^{T}}$, can also be further simplified as follows.

$$\mathbf{X_r'^{*}R_r^{(k)}\hat{Z}_r^{T}} = \begin{bmatrix} 1 & \cdots & 1 \\ 0 & \cdots & 0 \\ 0 & \cdots & 0 \end{bmatrix} \mathbf{R_r^{(k)}} \begin{bmatrix} \mathbf{\hat{Z}_{r[1,1]}} & \cdots & \mathbf{\hat{Z}_{r[d,1]}} \\ \vdots & \ddots & \vdots \\ \mathbf{\hat{Z}_{r[1,n]}} & \cdots & \mathbf{\hat{Z}_{r[d,n]}} \end{bmatrix}$$

$$= \begin{bmatrix} \mathbf{R_{r[k,1]}} & \cdots & \mathbf{R_{r[k,n]}} \\ 0 & \cdots & 0 \\ 0 & \cdots & 0 \end{bmatrix} \begin{bmatrix} \mathbf{\hat{Z}_{r[1,1]}} & \cdots & \mathbf{\hat{Z}_{r[d,1]}} \\ \vdots & \ddots & \vdots \\ \mathbf{\hat{Z}_{r[1,n]}} & \cdots & \mathbf{\hat{Z}_{r[d,n]}} \end{bmatrix}$$

$$= \begin{bmatrix} \mathbf{R_{r[k,\cdot]}} \cdot \mathbf{\hat{Z}^{T}_{r[\cdot,1]}} & \cdots & \mathbf{R_{r[k,\cdot]}} \cdot \mathbf{\hat{Z}^{T}_{r[\cdot,d]}} \\ 0 & \cdots & 0 \\ 0 & \cdots & 0 \end{bmatrix}$$

Therefore, to capture this term for all k clusters, we need only save $\mathbf{C} \in \mathbb{R}^{k\times d}$, a matrix containing k rows, where each row consists of the vector $\left[\mathbf{R_{r[k,\cdot]}} \cdot \mathbf{\hat{Z}^{T}_{r[\cdot,1]}} \quad \cdots \quad \mathbf{R_{r[k,\cdot]}} \cdot \mathbf{\hat{Z}^{T}_{r[\cdot,d]}}\right]$ for the corresponding cluster. We can directly calculate $\mathbf{C} = \mathbf{R_r\hat{Z}_r^{T}}$.

Supplementary References

1. Popescu, D.-M. *et al.* Decoding human fetal liver haematopoiesis. *Nature* **574**, 365–371 (2019).
2. Abdelaal, T. *et al.* A comparison of automatic cell identification methods for single-cell RNA sequencing data. *Genome Biol.* **20**, 194 (2019).
3. Ren, X. *et al.* COVID-19 immune features revealed by a large-scale single-cell transcriptome atlas. *Cell* **184**, 1895-1913.e19 (2021).
4. Baron, M. *et al.* A Single-Cell Transcriptomic Map of the Human and Mouse Pancreas Reveals Inter- and Intra-cell Population Structure. *Cell Syst* **3**, 346-360.e4 (2016).
5. Buenrostro, J. D. *et al.* Integrated Single-Cell Analysis Maps the Continuous Regulatory Landscape of Human Hematopoietic Differentiation. *Cell* vol. 173 1535-1548.e16 (2018).
6. Korsunsky, I. *et al.* Fast, sensitive and accurate integration of single-cell data with Harmony. *Nat. Methods* **16**, 1289–1296 (2019).
