# Supplementary material for: Efficient and precise single-cell reference atlas mapping with Symphony
Source: Nat Commun. 2021 Oct 7;12:5890. doi: 10.1038/s41467-021-25957-x (PMC8497570; doi:10.1038/s41467-021-25957-x)
Supplement: Supplementary file 3 — Reporting Summary [file 41467_2021_25957_MOESM3_ESM.pdf]

nature research

Corresponding author(s): Ilya Korsunsky, PhD
Soumya Raychaudhuri, MD, PhD

Last updated by author(s): 8/30/2021

Reporting Summary

Nature Research wishes to improve the reproducibility of the work that we publish. This form provides structure for consistency and transparency in reporting. For further information on Nature Research policies, see our Editorial Policies and the Editorial Policy Checklist.

Statistics

For all statistical analyses, confirm that the following items are present in the figure legend, table legend, main text, or Methods section.

n/a	Confirmed	
☐	☒	The exact sample size (*n*) for each experimental group/condition, given as a discrete number and unit of measurement
☐	☒	A statement on whether measurements were taken from distinct samples or whether the same sample was measured repeatedly
☐	☒	The statistical test(s) used AND whether they are one- or two-sided<br>*Only common tests should be described solely by name; describe more complex techniques in the Methods section.*
☐	☒	A description of all covariates tested
☐	☒	A description of any assumptions or corrections, such as tests of normality and adjustment for multiple comparisons
☐	☒	A full description of the statistical parameters including central tendency (e.g. means) or other basic estimates (e.g. regression coefficient) AND variation (e.g. standard deviation) or associated estimates of uncertainty (e.g. confidence intervals)
☐	☒	For null hypothesis testing, the test statistic (e.g. *F*, *t*, *r*) with confidence intervals, effect sizes, degrees of freedom and *P* value noted<br>*Give P values as exact values whenever suitable.*
☒	☐	For Bayesian analysis, information on the choice of priors and Markov chain Monte Carlo settings
☒	☐	For hierarchical and complex designs, identification of the appropriate level for tests and full reporting of outcomes
☐	☒	Estimates of effect sizes (e.g. Cohen's *d*, Pearson's *r*), indicating how they were calculated

Our web collection on statistics for biologists contains articles on many of the points above.

Software and code

Policy information about availability of computer code

Data collection	No software was used to collect the data (expression matrices and metadata was directly downloaded from public sources).
Data analysis	The code for the Symphony package is deposited on GitHub (https://github.com/immunogenomics/symphony) and is also available as an R package on CRAN. All notebooks and scripts used to generate figures and analyze the data are located at https://github.com/immunogenomics/symphony_reproducibility.<br><br>We used custom scripts for preprocessing the data. We used the following open source R packages for various analyses and visualization: ggrastr (v0.2.3), pheatmap (v1.0.12), RColorBrewer (v1.1-2), stringr (v1.4.0), ggplot2 (v3.3.5), irlba (v2.3.3), Matrix (v1.3-3), uwot (v0.1.10), harmony (v0.1.0), Seurat (v4.0.2), singlecellmethods (v0.1.0), presto (v1.0.0), lisi (v1.0), ggrepel (v0.9.1), biomaRt (v2.46.3), class (v7.3-19), RANN (v2.6.1), Rcpp (v1.0.6). Open source R script evaluate.R was downloaded from GitHub: https://github.com/tabdelaal/scRNAseq_Benchmark. We used the open source python packages: scArches (v0.3.0), scipy (v1.6.0), scanpy (1.7.2), pandas (v1.2.1), numpy (1.19.2).

For manuscripts utilizing custom algorithms or software that are central to the research but not yet described in published literature, software must be made available to editors and reviewers. We strongly encourage code deposition in a community repository (e.g. GitHub). See the Nature Research guidelines for submitting code & software for further information.

Data

Policy information about availability of data

All manuscripts must include a data availability statement. This statement should provide the following information, where applicable:

- Accession codes, unique identifiers, or web links for publicly available datasets
- A list of figures that have associated raw data
- A description of any restrictions on data availability

Datasets for all analyses were obtained from publicly available sources, for which the specific links are listed in Supplementary Table 1. Additionally, we provide a compendium of 8 pre-built Symphony references available for download on Zenodo (see Table 1 for links).
The 10x PBMCs data matrices were obtained from Korsunsky et al. (2019): https://github.com/immunogenomics/harmony2019/tree/master/data/figure4; original files from 10x Genomics: https://support.10xgenomics.com/single-cell-gene-expression/datasets. The pancreas reference data matrices were obtained from Korsunsky et al. (2019): https://github.com/immunogenomics/harmony2019/tree/master/data/figure5; original data is located on GEO (GSE81076, GSE85241, GSE86469) and EMBL-EBI (E-MTAB-5061). The human and mouse pancreas query data (Baron et al., 2016) was downloaded from https://hemberg-lab.github.io/scRNA.seq.datasets/human/pancreas. The fetal liver hematopoiesis data from Popescu et al. (2019) is located on EMBL-EBI (E-MTAB-7407), and post-doublet removal data was kindly provided by the authors. The PbmcBench data were obtained from the Zenodo repository for Abdelaal et al. (2019): https://zenodo.org/record/3357167#.YSL8p9NKhTY. The memory T cell CITE-seq dataset from Nathan et al. (2021) is available on GEO (GSE158769). The healthy fetal kidney data (Stewart et al., 2019) was obtained from https://www.kidneycellatlas.org/. The renal cell carcinoma data (Bi et al., 2021) was obtained from the Broad Institute Single Cell Portal (SCP1288). The 1.46 million cell COVID-19 dataset (Ren et al., 2021) is available on GEO (GSE158055), and .h5ad file was obtained from https://drive.google.com/file/d/1TXDJqOvFkJxbcm2u2-_bM5RBdTOqv56w/view. The scATAC-seq hematopoiesis dataset (Buenrostro et al., 2018) was downloaded from the Pinello Lab GitHub: https://github.com/pinellolab/scATAC- benchmarking/blob/master/Real_Data/Buenrostro_2018/input/combined.sorted.merged.bed.
Gencode .gtf files for versions 4-38 (used for determining gene name synonyms in cancer analysis) were downloaded from: http://ftp.ebi.ac.uk/pub/databases/gencode/Gencode_human.

Field-specific reporting

Please select the one below that is the best fit for your research. If you are not sure, read the appropriate sections before making your selection.

☒ Life sciences ☐ Behavioural & social sciences ☐ Ecological, evolutionary & environmental sciences

For a reference copy of the document with all sections, see nature.com/documents/nr-reporting-summary-flat.pdf

Life sciences study design

All studies must disclose on these points even when the disclosure is negative.

Sample size	None of our statistical tests required the predetermined calculation of sample size. In order to assess Symphony's performance in various settings of single-cell dataset size, we selected datasets ranging from a few thousand cells to 1.46 million cells. The sample sizes are sufficient because they are considered standard or large based on current capabilities of single-cell sequencing experiments.
Data exclusions	For the 10x PBMC and pancreatic islet cell datasets, we filtered out low-quality cells in the standard manner as described in Korsunsky et al. (Nat Methods, 2019) and Methods. For the fetal liver hematopoiesis dataset, the preprocessed and filtered version of the dataset with doublets removed was obtained by contacting the original authors (Popescu et al., Nature 2019); we excluded data from 2 donors (5'-sequenced data only) due to low library complexity (see Supplementary Fig. 7).
Replication	We demonstrate the performance of Symphony on a variety of real single-cell datasets, spanning many different scRNA-seq technologies and donors. For the 10x PBMC analyses, we replicate the reference building and mapping experiments with each of the 3 possible reference / query splits of the 3 datasets (yielding similar performance). To assess Symphony's performance in other biological contexts (e.g. different cell types) and across different single-cell sequencing technologies, we tested it in a variety of additional contexts using one predefined reference/query split per context with multiple donors represented in both reference and query. In all tested cases, we replicated Symphony's ability to build references and map queries accurately and efficiently. These additional examples include pancreas islet cells (mapping inDrops onto a plate-based reference), fetal liver hematopoiesis (mapping 10x 5' to 3'), and memory T cells (mapping a random subset of donors assayed using CITE-seq). For the fetal liver example, we additionally confirmed reproducibility internally using 14 "held-out donor" experiments within the 3' data alone (yielding similar performance; unpublished).
Randomization	N/A. This study did not involve experimental grouping.
Blinding	N/A. This study did not involve experimental grouping.

Reporting for specific materials, systems and methods

We require information from authors about some types of materials, experimental systems and methods used in many studies. Here, indicate whether each material, system or method listed is relevant to your study. If you are not sure if a list item applies to your research, read the appropriate section before selecting a response.

Materials & experimental systems

n/a	Involved in the study
☒	☐ Antibodies
☒	☐ Eukaryotic cell lines
☒	☐ Palaeontology and archaeology
☒	☐ Animals and other organisms
☒	☐ Human research participants
☒	☐ Clinical data
☒	☐ Dual use research of concern

Methods

n/a	Involved in the study
☒	☐ ChIP-seq
☒	☐ Flow cytometry
☒	☐ MRI-based neuroimaging
